# Glutathione peroxidase 3 preserves hepatocyte mitochondrial quality control to enhance macrophage pro‐regenerative phenotype during liver regeneration

**DOI:** 10.1002/ctm2.70695

**Published:** 2026-05-29

**Authors:** Yuechen Wang, Jian Xu, Zeyu Zhu, Ye Zhang, Haoran Hu, Yiyun Gao, Yuting Tao, Feifan Yao, Suiqing Zhou, Weizhe Zhong, Zhuqing Rao, Haoming Zhou, Xuehao Wang

**Affiliations:** ^1^ Hepatobiliary Center, The First Affiliated Hospital with Nanjing Medical University, Key Laboratory of Liver Transplantation, Chinese Academy of Medical Sciences, Key Laboratory of Hepatobiliary Tumors, National Health Commission, Jiangsu Provincial Medical Innovation Center, Jiangsu Provincial Medical Key Laboratory Nanjing Medical University Nanjing Jiangsu Province China; ^2^ Department of Anesthesiology and Perioperative Medicine The First Affiliated Hospital with Nanjing Medical University Nanjing China; ^3^ Collaborative Innovation Center for Cancer Personalized Medicine Nanjing Medical University Nanjing Jiangsu Province China

**Keywords:** liver regeneration, hepatocyte, mitochondrial quality control, macrophage, cGAS‐STING

## Abstract

**Background:**

Precise regulation of mitochondrial function is critical for liver regeneration. However, the underlying regulatory mechanism remains elusive. Here, we aimed to investigate the role of hepatocellular glutathione peroxidase 3 (GPX3) in liver regeneration.

**Methods:**

In a 70% partial hepatectomy (PH) mouse model, immunostaining and single‐cell RNA sequencing revealed significant enrichment but down‐regulation of mitochondrial oxidative phosphorylation pathways post‐PH, along with up‐regulated hypoxia‐inducible factor 1a (HIF‐1a) and GPX3 in hepatocytes. Single‐cell analysis confirmed peak GPX3 expression in hepatocytes at day 2 post‐PH. Hepatocyte‐specific GPX3 knockout impaired mitochondrial function and delayed liver regeneration.

**Results:**

Mechanistically, immunoprecipitation–mass spectrometry and MitoCarta3.0 analysis identified voltage‐dependent anion channel 1 (VDAC1) as a direct GPX3‐binding partner. GPX3 interacted with VDAC1 via its A2 domain (residues 75–150), suppressing VDAC1 oligomerisation to restore mitochondrial Ca^2+^ homeostasis and preserve mitochondrial quality control (MQC). Notably, GPX3 deficiency promoted mitochondrial DNA (mtDNA) release, activating the cyclic GMP–AMP synthase (cGAS)–stimulator of interferon genes (STING) pathway in macrophages. Persistent STING hyperactivation increased interferon production while suppressing hepatocyte growth factor release, further inhibiting regeneration. Critically, GPX3 overexpression enhanced liver regeneration in both PH and hepatic ischemia–reperfusion injury models, underscoring its central role across regenerative stressors.

**Conclusions:**

In conclusion, GPX3 promotes liver regeneration by inhibiting VDAC1 oligomerisation to stabilise mitochondrial Ca^2+^ dynamics and MQC, while preventing mtDNA‐mediated functional and phenotypic alterations in macrophages, positioning it as a therapeutic target for liver regeneration.

**Key points:**

- GPX3 directly binds VDAC1 via its A2 domain to suppress VDAC1 oligomerisation, restoring mitochondrial Ca^2^^+^ homeostasis and preserving mitochondrial quality control during liver regeneration.
- GPX3 deficiency promotes mtDNA release, hyperactivating the cGAS–STING pathway in macrophages and suppressing hepatocyte growth factor (HGF) release.
- GPX3 overexpression enhances liver regeneration in both partial hepatectomy and hepatic ischemia–reperfusion injury models, highlighting its therapeutic potential.

## Graphical abstract

(1) GPX3 inhibits VDAC1 oligomerisation by directly binding to it. (2) The loss of GPX3 leads to VDAC1 oligomerisation, resulting in dysregulation of mitochondrial quality control, characterised by enhanced fission, weakened fusion and impaired autophagy. (3) Concurrently, VDAC1 oligomerisation promotes mtDNA release into the cytosol, which ultimately activates the STING signalling pathway in macrophages

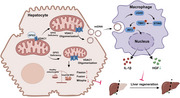

## 1 INTRODUCTION

The unique regenerative capacity of the liver is essential for recovery of liver function post‐partial hepatectomy (PH), living‐donor liver transplantation or chronic and acute liver injury.^1^, ^2^, ^3^ Impaired liver regeneration can result in liver dysfunction or even liver failure.^4^ However, the precise regulatory mechanism of liver regeneration remains elusive and the effective strategies to promote liver regeneration is still lacking, suggesting the importance of identification of the key molecular and cellular players.

Hepatocyte regeneration is one of the hallmarks of liver regeneration.^5^ Upon liver injury or liver tissue loss, quiescent hepatocytes re‐enter the cell cycle to proliferate rapidly to return the liver to equivalent size and weight to those prior to injury.^6^ Various extracellular and intracellular signalling pathways play an important role in regulating hepatocyte regeneration.^2^, ^7^ Studies using spatial transcriptomics have revealed functional heterogeneity among hepatocytes across liver zones, with Zone 2 hepatocytes identified as a major source of regeneration.^8^, ^9^ When hepatocyte proliferation is compromised, biliary epithelial cells can proliferate at low efficiency and transdifferentiate into hepatocytes.^2^ Of note, dysregulated proliferation of the surviving hepatocytes upon sustained exposure to genotoxic injury may cause neoplasia or cancer.^2^, ^10^ Therefore, precise regulation of hepatocellular proliferation is essential for optimal liver regeneration and liver function.

Mitochondria, the cellular powerhouses, are indispensable for liver regeneration.^11^, ^12^, ^13^, ^14^ Post‐liver resection, mitochondria support hepatocyte proliferation by generating ATP via oxidative phosphorylation (OXPHOS) to meet bioenergetic demands.^15^ Interestingly, it has been reported that OXPHOS is suppressed in the early phase of liver regeneration,^16^ during which specific regulatory mechanisms might be used to ensure adequate energy supply by mitochondria for the regenerative process. Mitochondrial quality control (MQC), which encompasses key processes like mitochondrial biogenesis, mitochondrial dynamics and mitophagy, is critical for stabilising mitochondrial function.^17^ However, whether and how MQC is regulated during liver regeneration remain poorly understood.

Macrophages represent one of the most abundant non‐parenchymal cells in the liver and play a significant role in liver injury and regeneration.^18^, ^19^ The cyclic GMP–AMP synthase (cGAS)–stimulator of interferon genes (STING) pathway is a key innate immune signalling cascade activated by cytosolic DNA.^20^ Our previous research demonstrated that aberrant STING activation in macrophages significantly impairs liver regeneration.^21^ As is well known, the cGAS–STING pathway is activated by mitochondrial DNA (mtDNA), and our study has found that GPX3 deficiency leads to mtDNA release. Nevertheless, whether hepatocyte GPX3 regulates these processes during liver regeneration remains unclear.

Glutathione peroxidase 3 (GPX3), an endogenous antioxidant enzyme, has been implicated in mitochondrial function.^22^ GPX3 functions in regulating mitochondrial redox and protein homeostasis through interacting with the Mia40.^23^ GPX3 also counteracts senescence in adipose remodelling by preserving mitochondrial homeostasis.^24^ Exogenous GPX3 delivery has been found to mitigate hepatocyte apoptosis and liver injury.^25^ Interestingly, PH causes hypoxia in liver tissues accompanied by elevated hypoxia‐inducible factor 1a (HIF‐1a) expression.^2^, ^26^, ^27^ Moreover, hypoxia has been shown to promote GPX3 gene induction via its specific binding site for HIF‐1.^28^ However, the precise role and mechanism of GPX3 in regulating MQC during liver regeneration remains unclear.

In this study, we identified significant up‐regulation of GPX3 expression in hepatocytes during liver regeneration. Utilising hepatocyte‐specific GPX3 knockout and overexpression mouse models, we observed that GPX3 directly binds to VDAC1 and modulates its oligomerisation, thereby enhancing mitochondrial function through the preservation of MQC in hepatocytes. Importantly, GPX3‐mediated regulation was further shown to prevent mtDNA‐induced functional and phenotypic perturbations in macrophages, highlighting its dual role in coordinating hepatocyte–macrophage crosstalk during liver regeneration.

## 2 MATERIALS AND METHODS

### 2.1 Animal studies

The wild‐type (WT), GPX3–FloxP (GPX3^fl/fl^), Alb‐Cre mice used in this study were obtained from GemPharmatech Co (Nanjing, Jiangsu, China). Hepatocyte‐specific GPX3 knockout (HKO) mice were generated by breeding GPX3–FloxP with Alb‐Cre mice. Briefly, homozygous GPX3^fl/fl^ mice were first bred with homozygous Alb‐Cre mice, and the heterozygous offspring (for both GPX3 and Cre) were back‐crossed with homozygous GPX3^fl/fl^ mice. This backcrossing strategy was used to generate GPX3^fl/fl^Alb^Cre^ mice together with GPX3^fl/fl^ littermate controls on a comparable genetic background.

All mice were kept under specific pathogen‐free conditions on a 12 h light/dark cycle, with free access to water and standard chow supplemented as required. Humane treatment of animals was ensured throughout, and all procedures were conducted in compliance with the relevant legal and ethical standards, following protocol number IACUC‐2311005 approved by the Institutional Animal Care and Use Committee of Nanjing Medical University.

### 2.2 Generation of mouse hepatectomy model

A mouse PH model was established to study liver regeneration. Briefly, male C57BL/6 mice (8–10 weeks old) were anesthetised with isoflurane, and a 70% PH was performed according to the standard method as described (PMID: 18600221). The left lateral and median lobes of the liver were carefully ligated and surgically removed under sterile conditions. Sham‐operated mice underwent the same surgical procedure without liver lobe resection. After surgery, mice were placed on a heating pad and monitored until full recovery. Liver tissues were collected at the indicated time points for further analysis.

### 2.3 Analysis of single‐cell RNA sequencing data

Publicly available scRNA‐seq datasets (CNP0002310) were obtained from the CNGBdb (China National GeneBank DataBase).^29^ Raw data were processed and analysed using the Seurat R package (v4.3.0) following standard workflows for quality control, normalisation and dimensionality reduction. Cell clusters were identified via tSNE and UMAP, with marker genes determined by differential expression analysis (Wilcoxon rank‐sum test, adjusted *p < .05).

### 2.4 Statistical analysis

All data were analysed using SPSS Statistics (version 19.0; SPSS, NY, USA) and GraphPad Prism 8.0 (GraphPad Software, CA, USA). Results are presented as mean ± SD or mean ± SEM from at least three independent experiments, as indicated in the figure legends. Normality was assessed using the Shapiro–Wilk test. For comparisons between two groups, a two‐tailed unpaired Student's *t* test was used for normally distributed data, whereas the Mann–Whitney test was used for non‐normally distributed data. For experiments involving a single factor with more than two groups, one‐way ANOVA followed by Tukey's multiple comparisons test was applied. For experiments involving two factors, such as genotype/treatment and time, two‐way ANOVA followed by Tukey's multiple comparisons test was used to assess the main effects and their interaction. Welch's ANOVA with Dunnett's T3 post hoc test was used when appropriate. The specific statistical methods used for each experiment are indicated in the corresponding figure legends. Statistical significance was considered at *p* < .05 (n.s. = not significant, *p < .05, **p < .01 and ***p < .001).

Details on other materials and methods are provided in the Supporting Information.

## 3 RESULTS

### 3.1 GPX3 expression is up‐regulated following PH

**FIGURE 1:**
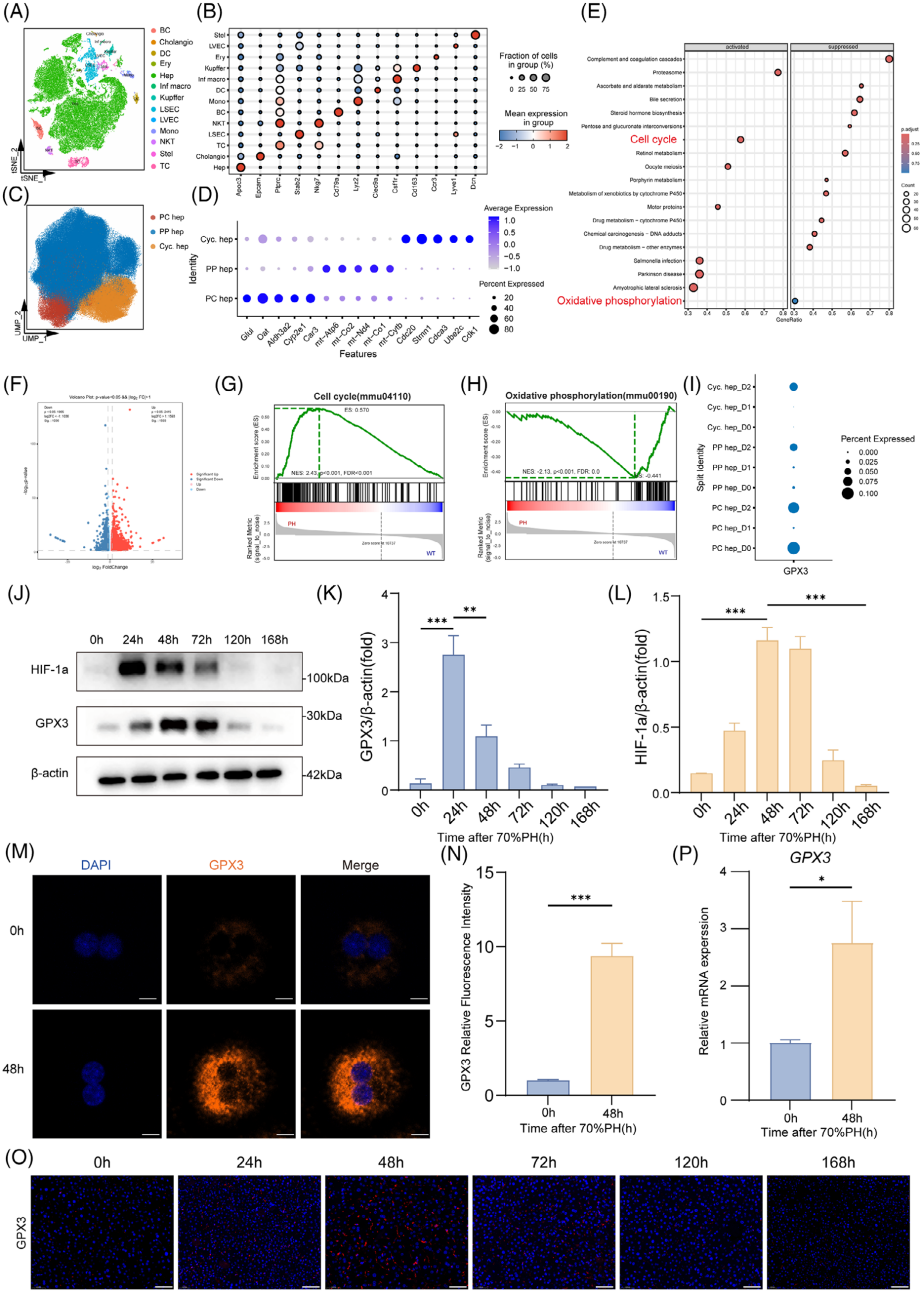
GPX3 expression is up‐regulated following partial hepatectomy. (A) t‐SNE plot showing the different subsets during liver regeneration. Colours represent different cell subsets. (B) Expression distribution of feature genes in each cell cluster. Dot size represents the proportion of cells expressing the gene, and colour indicates the average expression level. (C) UMAP visualisation showing the hepatocyte subsets. Colours represent different cell subsets. (D) Marker gene expression profiles of different hepatocyte subpopulations. (E) Pathway enrichment analysis of differentially expressed genes in hepatocytes. Dot size represents the number of genes, and colour indicates the significance of enrichment. (F) Volcano plot exhibiting gene expression log_2_ fold change between mice at 0 and 48 h post‐PH, *n* = 4. (G and H) GSEA showing significant enrichment of cell cycle pathway (G) and down‐regulation of oxidative phosphorylation pathway (H) in the PH group, *n* = 4. (I) Dot plot showing the expression of GPX3 for each hepatocyte subset. Colour represents post‐PH time points, and dot size indicates gene expression levels. (J–L) Western blot analysis and quantitation of GPX3 and HIF‐1a in primary hepatocytes at indicated time points post‐PH, *n* = 3. (M and N) Immunofluorescence staining revealed differential expression of GPX3 at 0 and 48 h after PH (scale bar = 10 µm). (O) Representative GPX3 immunohistochemical staining at indicated time points post‐PH (scale bar = 50 µm). (P) RNA levels of GPX3 measured by qRT‐PCR at 0 and 48 h after PH, *n* = 3. For experiments involving genotype/treatment and time, statistical significance was determined by two‐way ANOVA followed by Tukey's multiple comparisons test. For single‐time‐point comparisons, an unpaired two‐tailed Student's *t*‐test was used. *p < .05, **p < .01, ***p < .001.

We first analysed a public single‐cell RNA‐sequencing dataset of the hepatectomy model (CNP0002310)^29^ and performed dimensional reduction and cell clustering of the single‐cell transcriptomic data and annotated the major cell populations according to canonical marker genes (Figure 1A,B). Thirteen major cell types were identified, including hepatocytes, cholangiocytes, Kupffer cells, infiltrating macrophages, monocytes, liver sinusoidal endothelial cells, lymphatic vessel endothelial cells, stellate cells, dendritic cells and lymphocyte populations. Among these, hepatocytes formed a relatively compact cluster, indicating a well‐defined transcriptional identity. We next focused on hepatocytes and performed subclustering analysis, which identified three major hepatocyte subpopulations: periportal hepatocytes (PP Hep), pericentral hepatocytes (PC Hep) and cycling hepatocytes (Cyc. Hep) (Figure 1C,D). Specifically, the proportion of the Cyc. Hep subpopulation increased significantly on day 2 post‐PH, and this subpopulation exhibited high expression of cell cycle‐related genes, indicating that it is engaged in active cell cycle progression (Figure S1A). In addition, KEGG analysis of hepatocyte differentially expressed genes (DEGs) showed that cell cycle‐related pathways were significantly up‐regulated, whereas OXPHOS‐related pathways were suppressed during regeneration (Figure 1E). We further performed targeted GSEA and found that mitophagy‐related programs were enhanced, whereas mitochondrial biogenesis‐related programs were not obviously changed (Figure S1B). These findings suggest potential mitochondrial dysfunction and specific mechanisms to maintain mitochondrial homeostasis during liver regeneration after hepatectomy.

Then we established 70% PH on 8‐week‐old WT mice. Since 48 h is known to be the peak of liver regeneration,^2^, ^6^, ^18^ we collected liver tissues at 0 h (WT group) and 48 h post‐PH (PH group) for transcriptome sequencing (Figure S1C). RNA‐seq identified 2629 DEGs (fold change > 1.00, adjusted *p*‐value < .05), including 1593 significantly up‐regulated and 1036 down‐regulated genes (Figure 1F). GSEA revealed significant enrichment of cell cycle pathways in the PH group (Figure 1G), indicating active regeneration. Surprisingly, we also observed significant enrichment but down‐regulation of OXPHOS pathways in PH group (Figure 1H), consistent with the single‐cell data analysis described above.

Previous studies have indicated the regulatory role of GPX3 in mitochondrial function.^23^, ^24^ We then examined the expression pattern of GPX3 in the single‐cell dataset. Although GPX3 was not the most highly expressed gene in hepatocytes among all liver cell types, its expression was markedly up‐regulated in hepatocyte populations after PH (Figure S1D,E), with the most pronounced increase observed in the PP Hep and Cyc. Hep subpopulations (Figure 1I). Meanwhile, analysis of clinical portal vein embolisation (PVE) specimens revealed that GPX3 expression was significantly up‐regulated in post‐PVE liver tissues and positively correlated with liver regeneration‐related markers (Figure S2A–C). These findings suggest that hepatocyte GPX3 may have functional relevance during liver regeneration.

Simultaneously, we collected samples at 0, 24, 48, 72, 120 and 168 h post‐PH for Western blot analysis. Both GPX3 expression in liver tissue and primary hepatocytes gradually increased and peaked at 48–72 h (Figures 1J,K and S1F,G). Regarding the induction of HIF‐1a by hypoxia after hepatectomy and the known regulation of GPX3 by hypoxia,^26^, ^28^ we hypothesised that hypoxia would induce GPX3 expression via HIF‐1a during liver regeneration. Indeed, Western blot analysis revealed coordinated expression, with HIF‐1a protein levels peaking earlier at 24 h post‐PH, preceding the GPX3 peak at 48 h (Figure 1J–L). We further predicted potential binding sites between HIF‐1a and the GPX3 promoter using JASPAR and validated this interaction via chromatin immunoprecipitation assay (Figure S1H,I). The elevated expression of GPX3 at 48 h was further confirmed by cellular immunofluorescence (IF) and tissue fluorescence staining (Figure 1M–O). qPCR analysis confirmed significantly increased  GPX3 mRNA levels in liver tissues at 48 h post‐PH (Figure 1P). Collectively, these results demonstrate that GPX3 expression is induced in hepatocytes following PH, suggesting its potential involvement in regulating liver regeneration.

### 3.2 Hepatocyte‐specific GPX3 deficiency impairs liver regeneration

**FIGURE 2:**
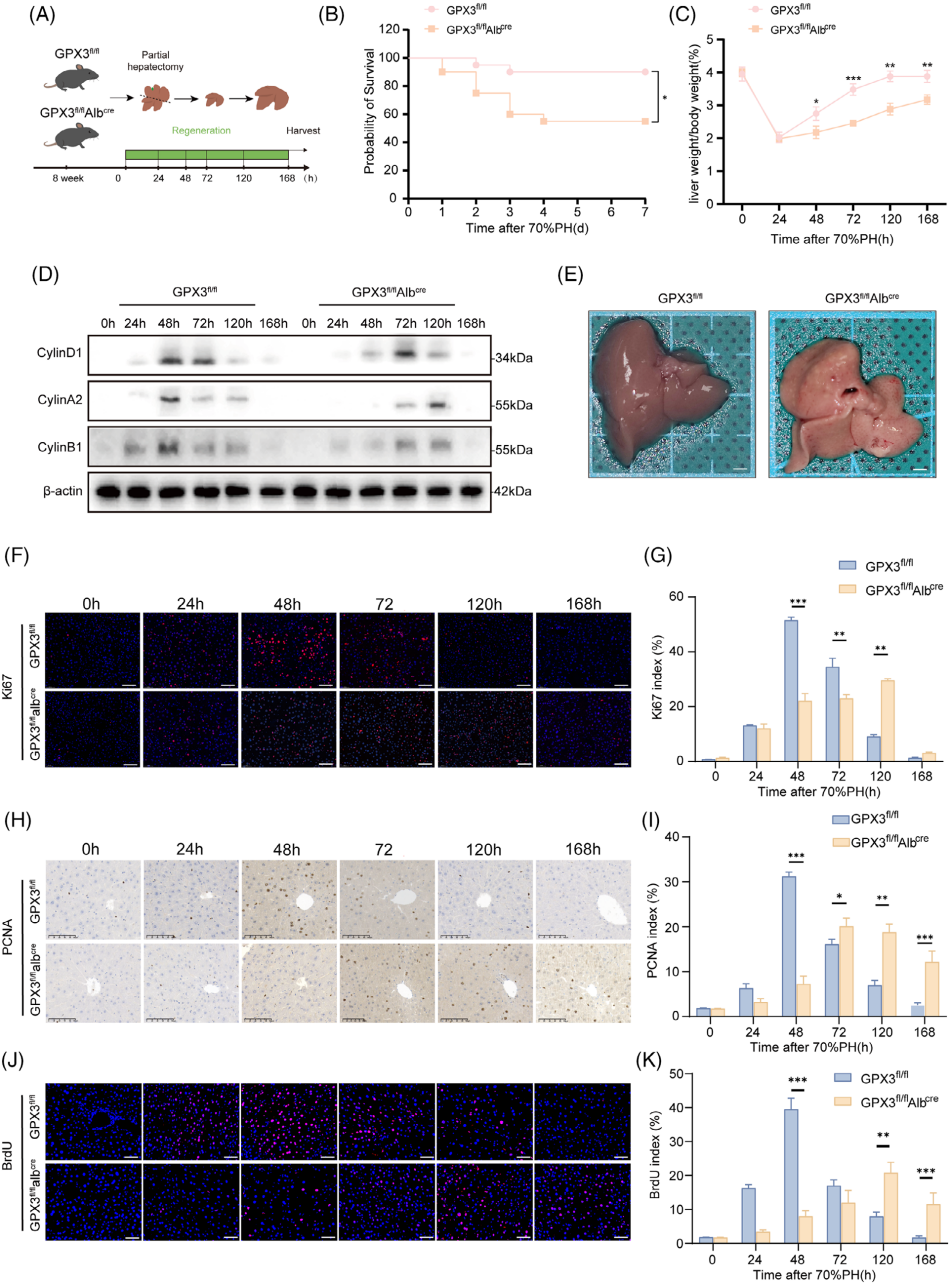
Hepatocyte‐specific GPX3 deficiency impairs liver regeneration. (A) Schematic overview of the standard 70% PH in mice, showing the resected liver lobes (dotted lines). (B) Survival of GPX3^fl/fl^Alb^cre^ and GPX3^fl/fl^ mice after 70%PH, *n* = 20/group. (C) The ratios of liver weight/body weight at different time points after PH. (D) Western blot analysis of cell cycle markers at different times after PH in GPX3^fl/fl^Alb^cre^ and GPX3^fl/fl^ mice, *n* = 3. (E) Liver morphology comparison between GPX3^fl/fl^Alb^cre^ and GPX3^fl/fl^ mice 48 h after PH. (F and G) Ki67 immunohistochemical staining in GPX3^fl/fl^Alb^cre^ and GPX3^fl/fl^ mice at various time points post‐PH (scale bar = 50 µm), with corresponding quantitative analysis shown on the right, *n* = 3. (H and I) PCNA immunohistochemical staining in GPX3^fl/fl^Alb^cre^ and GPX3^fl/fl^ mice at various time points post‐PH (scale bar = 100 µm), with corresponding quantitative analysis shown on the right, *n* = 3. (J and K) BrdU immunohistochemical staining in GPX3^fl/fl^Alb^cre^ and GPX3^fl/fl^ mice at various time points post‐PH (scale bar = 50 µm), with corresponding quantitative analysis shown on the right, *n* = 3. For experiments involving genotype/treatment and time, statistical significance was determined by two‐way ANOVA followed by Tukey's multiple comparisons test. For single‐time‐point comparisons, an unpaired two‐tailed Student's *t*‐test was used. *p < .05, **p < .01, ***p < .001.

To further evaluate the role of GPX3 in liver regeneration, hepatocyte‐specific GPX3 knockout mice (GPX3^fl/fl^Alb^cre^) and their littermate controls (GPX3^fl/fl^) were generated followed by PH model (Figures 2A and S3A,B). We validated GPX3 expression in knockout mice by Western blot and qPCR (Figure S3C,D). Interestingly, GPX3^fl/fl^Alb^cre^ mice exhibited lower survival rates compared with GPX3^fl/fl^ mice (Figure 2B). Meanwhile, as compared with littermate controls, GPX3^fl/fl^Alb^cre^ mice showed significantly lower liver‐to‐body weight ratio and failed to fully recover after 7 days (Figure 2C) with more severe liver injury as indicated by ALT and AST (Figure S4A,B) and HE staining analysis at 48 h post‐PH (Figure S4C). Despite a relative late‐phase increase in some proliferation markers, GPX3‐deficient mice exhibited persistently impaired liver‐to‐body weight recovery and more severe injury, indicating delayed compensatory regeneration.

Cell proliferation of hepatocytes was examined as well and revealed that GPX3 depletion persistently and significantly suppressed Cyclin D1 (a G1 phase marker), Cyclin A2 (functions in G1/S phase transition) and Cyclin B1 (specifically expressed during G2/M phase) expression in hepatocytes during liver regeneration (Figure 2D). GPX3‐deficient livers also exhibited more severe inflammation at 48 h (Figure 2E). Immunohistochemistry staining of liver tissues further demonstrated that Ki67, PCNA and BrdU positive hepatocytes were significantly reduced in GPX3^fl/fl^Alb^cre^ mice (Figure 2F–K). These results indicated that GPX3 knockout suppressed hepatocyte proliferation and delayed liver regeneration.

### 3.3 GPX3 maintains hepatocyte mitochondrial function during liver regeneration

Mitochondrial function is closely associated with liver regeneration. Our study revealed impaired mitochondrial OXPHOS during liver regeneration (Figure 1E,H), suggesting that modulating mitochondrial function may represent a critical therapeutic target for regulating liver regeneration. Previous studies have reported GPX3's regulatory effects on mitochondria,^23^, ^24^, ^30^ prompting us to investigate whether GPX3 deficiency affects mitochondrial function in hepatocytes during liver regeneration.

**FIGURE 3:**
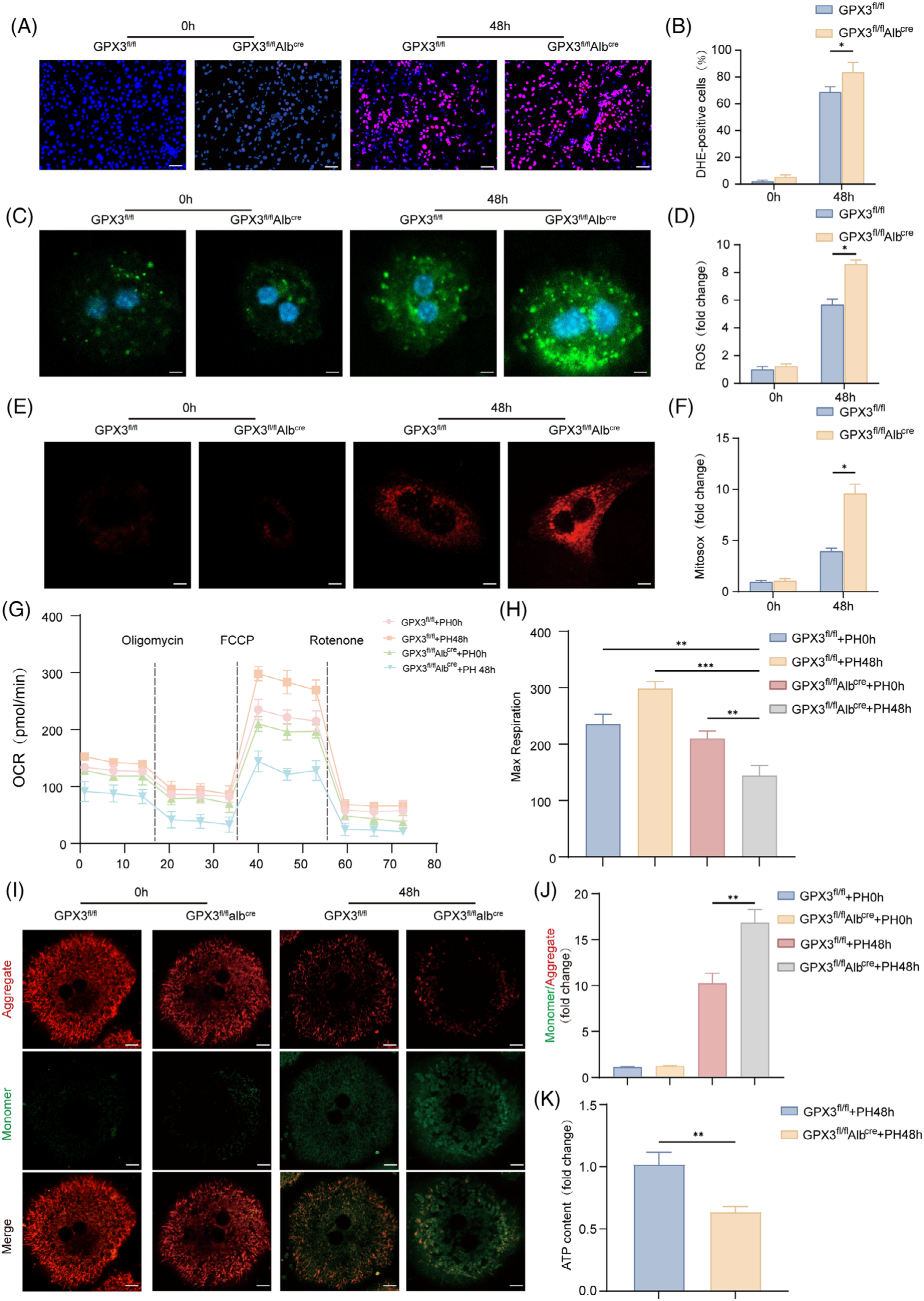
GPX3 maintains hepatocyte mitochondrial function during liver regeneration. (A and B) Left: DHE staining showing ROS levels in GPX3^fl/fl^Alb^cre^ and GPX3^fl/fl^ livers at 0 and 48 h post‐PH (scale bar = 50 µm). Right: Quantified DHE^+^ cells per field, *n* = 3. (C and D) Left: DCFH‐DA fluorescence imaging of primary hepatocytes showing ROS leve at 0 and 48 h post‐PH in GPX3^fl/fl^Alb^cre^ versus GPX3^fl/fl^ mice (scale bar = 10 µm). Right: Quantified ROS levels expressed as fold change relative to 0 h control, *n* = 3. (E and F) Left: MitoSOX Red fluorescence indicating mitochondrial superoxide production at 0 and 48 h post‐PH (scale bar = 10 µm). Right: Quantified fold change of MitoSOX signal intensity, *n* = 3. (G and H) OCR measurement in primary hepatocytes isolated from GPX3^fl/fl^Alb^cre^ and GPX3^fl/fl^ mice at 0 and 48 h post‐PH, and the analysis of mitochondrial respiration, *n* = 5. (I and J) Representative JC‐10‐stained images (I) and quantification of mitochondria depolarisation (J) in primary hepatocytes (scale bar = 10 µm), *n* = 3. (K) ATP content in primary hepatocytes isolated from GPX3^fl/fl^Alb^cre^ and GPX3^fl/fl^ mice at 0 and 48 h post‐PH, *n* = 5. For experiments involving genotype/treatment and time, statistical significance was determined by two‐way ANOVA followed by Tukey's multiple comparisons test. For single‐time‐point comparisons, an unpaired two‐tailed Student's *t*‐test was used. *p < .05, **p < .01, ***p < .001.

We first performed dihydroethidium staining on liver tissues at 0 and 48 h, which demonstrated significantly enhanced oxidative stress in GPX3^fl/fl^Alb^cre^ mice (Figure 3A,B). Subsequent isolation and IF analysis of primary hepatocytes revealed increased intracellular ROS following PH, with GPX3^fl/fl^Alb^cre^ mice exhibiting substantially higher ROS levels at 48 h post‐PH (Figure 3C,D). These findings indicate that GPX3 deficiency exacerbates oxidative stress in hepatocytes, suggesting potential mitochondrial dysfunction.

Further evaluation using MitoSOX Red confirmed enhanced mitochondrial superoxide production post‐PH, with GPX3 deficiency significantly amplifying this effect (Figure 3E,F). Seahorse analyser measurements demonstrated that hepatocyte‐specific GPX3 deletion markedly aggravated PH‐induced impairment of mitochondrial respiratory function (Figure 3G,H), accompanied by reduced cellular ATP levels in GPX3^fl/fl^Alb^cre^ mice (Figure 3K). JC‐10 staining revealed severe disruption of mitochondrial membrane potential in GPX3‐deficient hepatocytes at 48 h post‐PH (Figure 3I,J).

Collectively, these data demonstrate that GPX3 deficiency leads to severe mitochondrial dysfunction in hepatocytes during liver regeneration.

### 3.4 GPX3 deficiency disrupts MQC in hepatocytes

**FIGURE 4:**
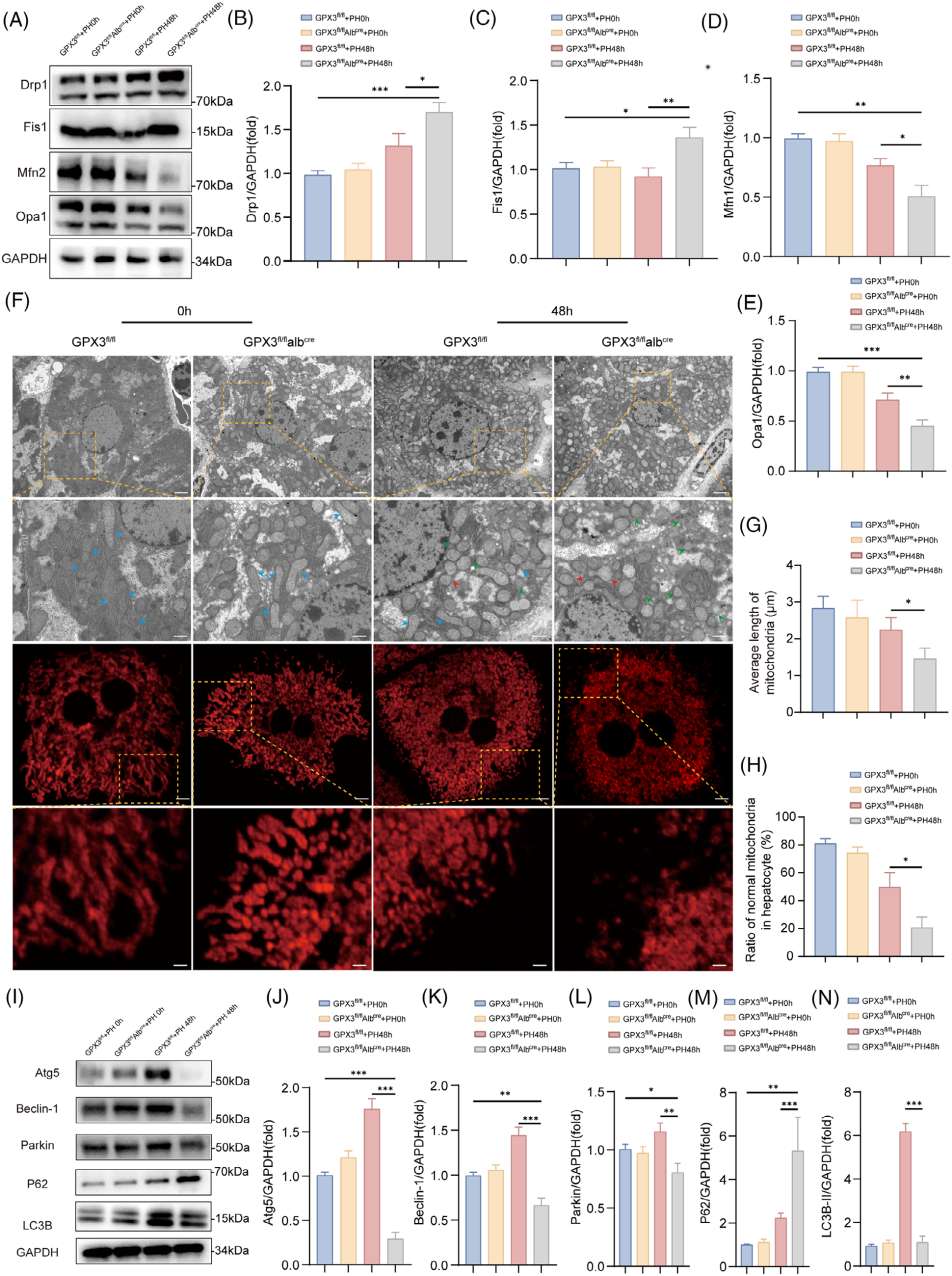
GPX3 deficiency disrupts MQC in hepatocytes. (A–E) Protein levels of Drp1, Fis1, Mfn2 and Opa1 in the primary hepatocytes. Statistics of protein grey relative to GAPDH were shown on the right, *n* = 3. (F) TEM (scale bar = 2 µm) and MitoTracker (scale bar = 10 µm) visualisation of hepatocyte mitochondria at 0 and 48 h post‐PH (blue arrows indicate normal mitochondria; red arrows indicate mitochondria undergoing fission; green arrows indicate mitochondria after fission). (G and H) Average mitochondrial length and the ratio of fragmented to tubular mitochondria were determined based on TEM, *n* = 5. (I–N) Protein levels of Atg5, Beclin‐1,  Parkin, P62 and LC3B in the primary hepatocytes. Statistics of protein grey relative to GAPDH were shown on the right, *n* = 3. For experiments involving genotype/treatment and time, statistical significance was determined by two‐way ANOVA followed by Tukey's multiple comparisons test. For single‐time‐point comparisons, an unpaired two‐tailed Student's *t*‐test was used. *p < .05, **p < .01, ***p < .001.

MQC represents a core mechanism for maintaining mitochondrial functional homeostasis and plays critical roles in the pathogenesis of various diseases.^17^ To investigate whether GPX3 affects hepatocyte mitochondrial homeostasis during PH by preserving MQC, we examined mitochondrial fission/fusion and mitophagy in primary hepatocytes after PH. Western blot analysis of primary hepatocytes showed that at 48 h post‐PH, GPX3^fl/fl^Alb^cre^ mice displayed significant up‐regulation of fission‐related proteins (DRP1 and FIS1) and marked down‐regulation of fusion‐related proteins (MFN2 and OPA1) compared with controls (Figure 4A–E), suggesting GPX3 deficiency disrupts the balance between mitochondrial fusion and fission following PH.

Further evaluation of mitochondrial morphology by transmission electron microscopy and MitoTracker staining revealed that GPX3^fl/fl^Alb^cre^ mice exhibited more small, round and fragmented mitochondria compared with GPX3^fl/fl^ mice (Figure 4F–H), with significantly reduced mitochondrial length (Figure 4G). Consistent with the above protein results of mitochondrial fission and fusion, these data indicated enhanced fission and impaired fusion in GPX3‐deficient hepatocytes.

The PINK1–Parkin pathway serves as a crucial regulator of mitophagy.^31^ Western blot analysis demonstrated that GPX3^fl/fl^ mice showed significant up‐regulation of Parkin expression post‐PH, accompanied by increased levels of Atg5, Beclin1 and LC3B‐II(Figure 4I–L), suggesting enhanced mitophagy as a potential protective mechanism after hepatectomy. Surprisingly, GPX3^fl/fl^Alb^cre^ mice exhibited significantly down‐regulated expression of Parkin, Atg5, Beclin1 and LC3B‐II, together with accumulation of P62 at 48 h post‐PH (Figure 4I–L), indicating suppressed mitophagy. To further evaluate mitophagy more directly, we performed Lv–mtKeima–COX8 staining in primary hepatocytes (Figure S5A). Consistent with the above findings, mtKeima analysis showed that mitophagy was significantly reduced in GPX3‐deficient hepatocytes after PH. These findings collectively demonstrate that GPX3 deficiency leads to impaired MQC.

### 3.5 GPX3 directly interacts with downstream protein VDAC1

**FIGURE 5:**
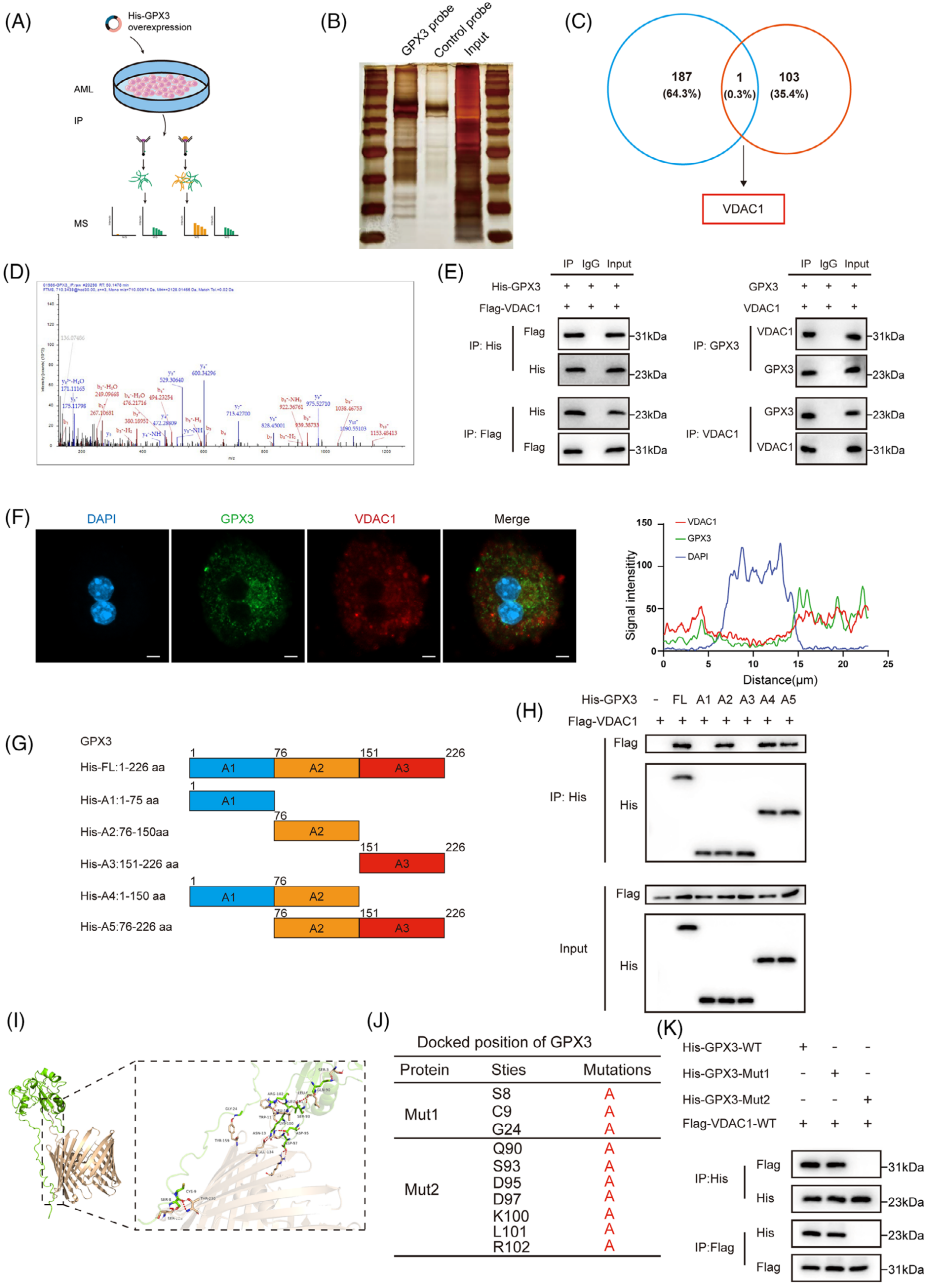
GPX3 directly interacts with downstream protein VDAC1. (A) GPX3 downstream target discovery via IP/MS. (B) Silver‐stained SDS‐PAGE gel‐containing proteins derived from immunoprecipitation by GPX3 and negative control, *n* = 3. (C) Venn analysis with results from MitoCarta3.0 and IP/MS. (D)Tandem mass spectrum identifying VDAC1 peptide. (E) Two‐way Co‐IP assays demonstrating the binding between GPX3 and VDAC1 were performed in NIH/3T3 cells (left) and primary hepatocytes after PH modelling (right), *n* = 3. (F) Representative images of immunofluorescence to verify the colocalisation of endogenous GPX3 and VDAC1 in primary hepatocytes, *n* = 3. Line graph showing quantitative assessment of the colocalisation of GPX3 and VDAC1 (scale bar = 10 µm). (G) Schematic overview of GPX3 in full‐length and truncations. (H) Immunoprecipitation and WB analyses illustrating interactions between His‐tagged truncated GPX3 and FLAG‐tagged truncated VDAC1 proteins in NIH/3T3 cells, *n* = 3. (I) Predicted binding model of GPX3 and VDAC1. (J) Docked positions of GPX3 and VDAC1 and mutations of TGPX3 interaction sites. (K) Immunoprecipitation and WB analyses showing interactions between His‐tagged mutated GPX3 and VDAC1 in NIH/3T3 cells, *n* = 3.

Based on previous findings regarding the regulatory role of GPX3 in mitochondrial proteins, we sought to further explore the downstream proteins regulated by GPX3 during PH. We performed immunoprecipitation coupled with mass spectrometry (IP/MS) on AML12 cells and identified 188 potential candidate proteins, which were further validated by silver staining (Figure 5A,B). To identify specific targets through which GPX3 affects MQC, we compared the genes encoding the potential interacting proteins obtained from IP/MS with the known mitochondrial dynamics and surveillance genes in the MitoCarta3.0 database (Figure 5C). This analysis led to the identification of VDAC1 as a downstream protein (Figure 5), which not only interacts with GPX3 but also participates in MQC.

To confirm the protein–protein interaction between GPX3 and VDAC1, His‐tagged GPX3 and FLAG‐tagged VDAC1 were overexpressed in NIH/3T3 cells. Co‐immunoprecipitation (Co‐IP) experiments revealed a reciprocal interaction: GPX3 co‐immunoprecipitated with VDAC1, and VDAC1 co‐immunoprecipitated with GPX3 (Figure 5E). Meanwhile, to verify whether this interaction exists under regenerative conditions, we performed endogenous Co‐IP experiments using primary hepatocytes isolated at 48 h post‐PH and obtained the same conclusion (Figure 5E). To further validate the endogenous interaction between GPX3 and VDAC1, IF analysis showed prominent co‐localisation of GPX3 and VDAC1 in the cytoplasm of mouse primary hepatocytes (Figure 5F).

To determine the specific regions involved in this protein–protein interaction, we designed a series of truncated mutants guided by protein structural analysis (Figure 5G,H). Co‐IP experiments performed in NIH/3T3 cells indicated that the A2 domain (residues 75–150) of GPX3 is essential for the GPX3–VDAC1 interaction (Figure 5I). We further predicted specific amino acid residues involved in the binding interaction using computational modelling via Haddock (Figure 5J). Subsequently, we mutated the predicted binding site between GPX3 and VDAC1, and Co‐IP analysis confirmed that the interaction between GPX3 and VDAC1 was effectively disrupted by the specific mutation of the predicted binding site (MUT2) (Figure 5K). These findings validate the interaction between GPX3 and VDAC1 and suggest that GPX3 may influence MQC through VDAC1, providing insights into the mechanistic regulation of MQC during hepatic regeneration.

### 3.6 VDAC1 oligomerisation modulated by GPX3 disrupts mitochondrial Ca^2^^+^ homeostasis and MQC

**FIGURE 6:**
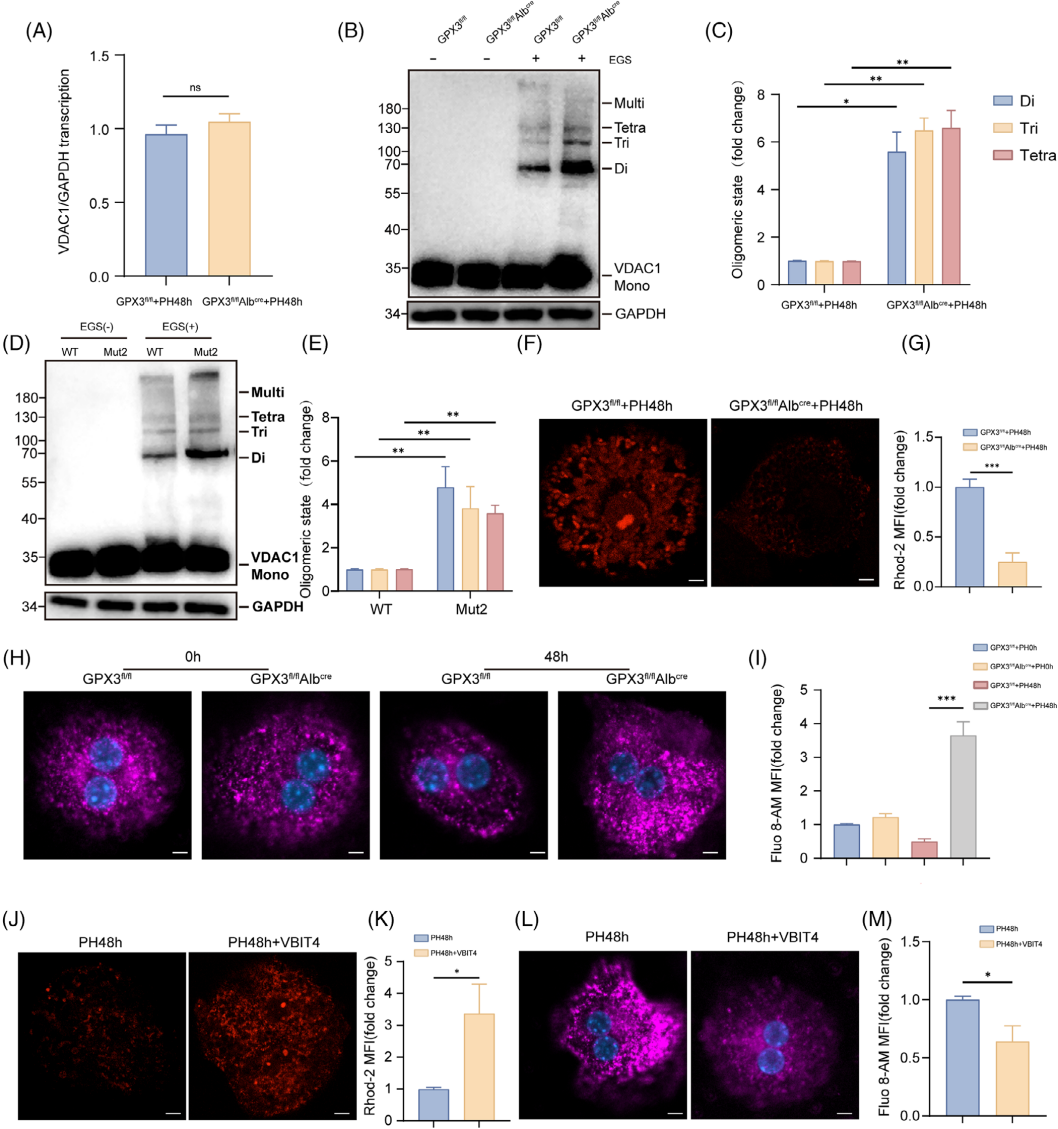
VDAC1 oligomerisation modulated by GPX3 disrupts mitochondrial Ca^2^^+^ homeostasis and MQC. (A) RNA levels of VDAC1 measured by qRT‐PCR at 0 and 48 h after PH, *n* = 3. (B and C) Western blot analysis of VDAC1 oligomerisation in primary hepatocytes from GPX3^fl/fl^Alb^cre^ and GPX3^fl/fl^ mice at 48 h post‐PH after treatment with the cross‐linking reagent ethylene glycol bis‐(succinimidyl succinate) (EGS) to stabilise the oligomers during electrophoresis, *n* = 3. (D and E) Western blot analysis of VDAC1 oligomerisation in NIH/3T3 cells with overexpressed GPX3 and MUT2 after treatment with EGS, *n* = 3. (F and G) Mitochondrial Ca^2^^+^ levels were measured using Rhod‐2 staining in primary hepatocytes (scale bar = 10 µm). Quantified Rhod‐2 staining intensity on the right, *n* = 3. (H and I) Cytosolic Ca^2^^+^ levels were measured using Fluo‐8 staining in primary hepatocytes (scale bar = 10 µm). Quantified Fluo‐8 staining intensity on the right, *n* = 3. (J and K) Following VIBT4 treatment, mitochondrial Ca^2^^+^ levels were assessed using Rhod‐2 staining. Quantified Rhod‐2 staining intensity on the right, *n* = 3. (L and M) Following VIBT4 treatment, cytosolic Ca^2^^+^ levels were assessed using Fluo‐8 staining. Quantified Fluo‐8 staining intensity on the right, *n* = 3. For experiments involving genotype/treatment and time, statistical significance was determined by two‐way ANOVA followed by Tukey's multiple comparisons test. For single‐time‐point comparisons, an unpaired two‐tailed Student's *t*‐test was used. *p < .05, **p < .01, ***p < .001.

VDAC1, located on the outer mitochondrial membrane (OMM), is a critical regulator of MQC. The oligomeric form of VDAC1 plays a pivotal role in maintaining mitochondrial function and overall cellular viability.^32^ To further investigate how GPX3 modulates VDAC1, we first examined the transcriptional levels of VDAC1 in GPX3^fl/fl^Alb^cre^ and GPX3^fl/fl^ mice following PH. No significant difference in VDAC1 mRNA expression was observed between the two groups (Figure 6A), suggesting that GPX3 does not regulate VDAC1 at the transcriptional level.

We then analysed VDAC1 protein oligomerisation by Western blot in primary hepatocytes post‐PH and found that GPX3‐deficient mice exhibited markedly increased VDAC1 oligomerisation (Figure 6B,C). To determine whether GPX3 regulates VDAC1 oligomerisation through direct interaction, we overexpressed His‐tagged WT GPX3 or the interaction‐deficient mutant GPX3 (MUT2) in NIH/3T3 cells and used LPS stimulation to induce VDAC1 oligomerisation. Notably, expression of GPX3–MUT2 significantly enhanced VDAC1 oligomer formation (Figure 6D,E), indicating that GPX3 suppresses VDAC1 oligomerisation via direct binding.

Considering that GPX3 functions as an antioxidant enzyme, its deficiency may lead to elevated ROS levels, which can induce VDAC1 oligomerisation.^33^ To distinguish between the antioxidant function of GPX3 and its direct VDAC1‐binding function, we treated GPX3‐deficient mice with the ROS scavenger N‐acetylcysteine (NAC). The results showed that NAC treatment significantly reduced ROS levels in liver tissues (Figure S6A) but only partially alleviated VDAC1 hyper‐oligomerisation (Figure S6B). Moreover, the mitochondrial membrane potential was not fully restored to normal levels (Figure S6C). These findings suggest that GPX3 may possess a structural function independent of its enzymatic activity.

Given that VDAC1 serves as a major mitochondrial Ca^2^^+^ channel and Ca^2^^+^ signalling plays an essential role in MQC regulation,^34^, ^35^ we next assessed intracellular Ca^2^^+^ distribution. Using Rhod‐2 staining, we observed a significant reduction in mitochondrial Ca^2^^+^ levels in hepatocytes from GPX3^fl/fl^Alb^cre^ mice after PH (Figure 6F,G). In contrast, Fluo‐8 staining revealed an increase in cytosolic Ca^2^^+^ levels in these mice (Figure 6H,I), suggesting a disruption in Ca^2^^+^ compartmentalisation associated with GPX3 deficiency. To determine whether this phenotype is directly attributable to increased VDAC1 oligomerisation, we treated mice with the VDAC1 oligomerisation inhibitor VBIT‐4^36^ (Figure S7A). VBIT‐4 administration restored mitochondrial Ca^2^^+^ levels and reduced cytosolic Ca^2^^+^ levels in GPX3^fl/fl^Alb^cre^ mice post‐PH (Figure 6J–M), effectively rescuing the Ca^2^^+^ distribution phenotype caused by GPX3 loss.

Collectively, these findings demonstrate that GPX3 deficiency leads to enhanced VDAC1 oligomerisation, which in turn disrupts mitochondrial Ca^2^^+^ homeostasis, highlighting a novel mechanism by which GPX3 preserves MQC during liver regeneration.

### 3.7 Suppression of VDAC1 oligomerisation abrogated the detrimental effects of hepatocyte GPX3 depletion during liver regeneration

To further investigate whether GPX3 deficiency affects MQC and ultimately impairs mitochondrial function and liver regeneration via modulation of VDAC1 oligomerisation, we administered the VDAC1 oligomerisation inhibitor VBIT‐4 in GPX3^fl/fl^Alb^cre^ mice following PH (Figure S7A). Strikingly, VBIT‐4 treatment restored mitochondrial morphology and length in GPX3‐deficient hepatocytes (Figure S8A–C). This morphological rescue was accompanied by reduced expression of fission‐related proteins, increased levels of fusion‐related proteins and up‐regulation of mitophagy markers (Figure S7B).

Assessment of mitochondrial membrane potential using JC‐10 dye revealed that VBIT‐4 restored the impaired mitochondrial membrane potential in GPX3^fl/fl^Alb^cre^ hepatocytes (Figure S8D,E). Furthermore, mitochondrial functional assays demonstrated improved oxygen consumption rate (OCR), enhanced maximal respiratory capacity and increased ATP production upon VBIT‐4 administration (Figure S8F–H). MitoSOX Red staining indicated reduced mitochondrial ROS, suggesting attenuated oxidative stress (Figure S8I). These findings collectively demonstrate that GPX3 deficiency impairs MQC and mitochondrial function through enhanced VDAC1 oligomerisation, and that pharmacological inhibition of VDAC1 oligomerisation by VBIT‐4 effectively rescues these defects.

To evaluate the impact of VDAC1 oligomerisation on liver regeneration, we assessed hepatocellular proliferation following PH. VBIT‐4 treatment significantly increased hepatic PCNA expression at 48 h post‐PH (Figure S8J, L) and rescued the delayed cyclin expression caused by GPX3 deficiency (Figures S7C and S8M), indicating enhanced regenerative activity. Meanwhile, VBIT‐4 treatment significantly increased the proportion of Ki67‐positive cells (Figure S8K).

Given the potential non‐specific effects of VBIT‐4 on VDAC1 oligomerisation inhibition,^37^ we further employed VBIT‐12, another VDAC1 oligomerisation inhibitor, to validate the role of GPX3 in modulating VDAC1.^38^ Notably, VBIT‐12 treatment also significantly suppressed VDAC1 hyper‐oligomerisation induced by GPX3 deficiency (Figure S9A), accompanied by decreased expression of fission‐related proteins, increased levels of fusion‐related proteins, up‐regulation of mitophagy markers and reduced mitochondrial ROS levels (Figure S9B,C). Moreover, VBIT‐12 treatment similarly rescued the impaired liver regeneration, as evidenced by increased PCNA expression and Ki67‐positive cell proportion (Figure S9D). Taken together, these results suggest that GPX3 promotes liver regeneration by restraining VDAC1 oligomerisation, thereby preserving MQC and mitochondrial function.

### 3.8 VDAC1 oligomerisation induces hepatocyte mtDNA release and activates cGAS–STING pathway in macrophages

**FIGURE 7:**
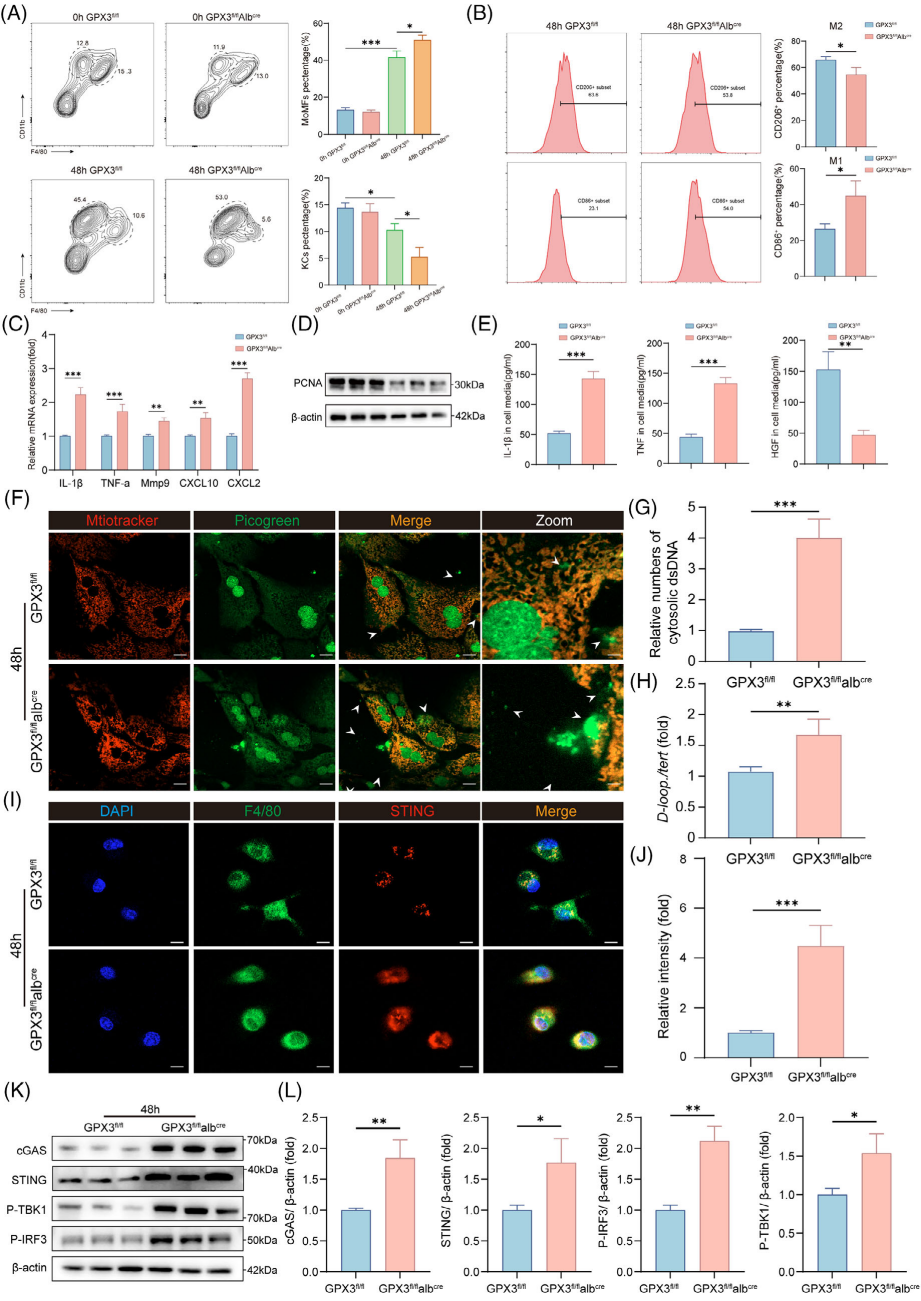
VDAC1 oligomerisation induces hepatocyte mtDNA release and activates cGAS–STING pathway in macrophages. (A) Representative FACS plots and quantification of MDMs and KCs in GPX3^fl/fl^Alb^cre^ and GPX3^fl/fl^ mice liver tissues 0 and 48 h after PHx (*n* = 6). (B) Representative flow cytometry histograms and quantification of CD206^+^ and CD86^+^ hepatic macrophages from GPX3^fl/fl^Alb^cre^ and GPX3^fl/fl^ mice at 48 h after PH. (C) QPCR analysis of inflammation‐related genes expression in MDMs in GPX3^fl/fl^Alb^cre^ at 48 h post‐PH, *n* = 3. (D) Western blot analysis of PCNA in hepatocytes from the co‐culture system, *n* = 3. (E) ELISA analysis of IL‐1β, TNF‐a and HGF secreted by macrophages from the co‐culture system, *n* = 3. (F and G) MtDNA released from mitochondria in GPX3^fl/fl^Alb^cre^ and GPX3^fl/fl^ mice post‐PH, as shown by confocal microscopy. Arrowheads, mtDNA released into cytoplasm (scale bar = 100 µm). (H) Relative amounts of total cytosolic mtDNA in macrophages isolated from the different groups were determined using qPCR with primers specific for mtDNA (D‐loop) and nuclear DNA (Tert), *n* = 3. (I and J) Intrahepatic macrophages were isolated from livers post‐PH followed by staining of STING (red), F4/80 (green) and nucleus (blue) (scale bar = 100 µm). (K and L) Western blot analysis and quantitation of cGAS, STING, p‐TBK1 and p‐IRF3, *n* = 3. For experiments involving genotype/treatment and time, statistical significance was determined by two‐way ANOVA followed by Tukey's multiple comparisons test. For single‐time‐point comparisons, an unpaired two‐tailed Student's *t*‐test was used. *p < .05, **p < .01, ***p < .001.

As a key non‐parenchymal cell population in the liver, macrophages play an essential role in liver regeneration.^18^ Studies have shown that the number of Kupffer cells decreases on days 1–2 after PH, while the number of monocyte‐derived macrophages (MDMs) increases significantly.^39^ Single‐cell data analysis revealed an increase in hepatic MDMs after PH (Figure S10A,B). Our analysis of intrahepatic macrophages collected at 48 h post‐PH revealed a significant increase in MDMs (Figure 7A), and GPX3 deficiency led to a further increase in MDMs. Meanwhile, we found that GPX3 deficiency resulted in a further decrease in M2‐like macrophages (Figure 7B). To further investigate the impact of GPX3 deficiency on macrophage function, we co‐cultured MDMs collected after PH with primary hepatocytes. The results showed that, although single‐cell analysis did not reveal significant enrichment of inflammatory genes in macrophages (Figure S10C,D), the co‐culture system from GPX3‐deficient mice exhibited significantly increased levels of inflammatory factors, up‐regulated expression of macrophage inflammation‐related genes and down‐regulated expression of hepatocyte proliferation‐related markers (Figure 7C–E). These findings indicate that GPX3 deficiency induces a phenotypic shift in macrophages, thereby inhibiting liver regeneration.

mtDNA leakage is a major consequence of mitochondrial dysfunction,^40^ and a study identifies that the formation of VDAC oligomers leads to the release of mtDNA.^36^ We therefore examined changes in mtDNA in GPX3^fl/fl^Alb^cre^ hepatocytes post‐PH. Unexpectedly, we observed increased mtDNA release from hepatocytes of GPX3‐deficient mice (Figure 7F,G), a finding further validated by qPCR results (Figure 7H). Studies indicate that extracellular mtDNA released from parenchymal cells serves as a critical upstream ligand activating the cGAS/cGAMP/STING signalling pathway.^41^ Concurrently, our group's prior research identified aberrant macrophage STING activation as a significant factor impairing liver regeneration.^21^ Consequently, we sought to investigate whether GPX3 regulates liver regeneration by modulating macrophage function and phenotype.

Macrophages isolated from GPX3 knockout mice exhibited enhanced activation of the STING signalling pathway, evidenced by increased STING staining (Figure 7I,J) and elevated protein levels of cGAS, STING, p‐TBK1 and p‐IRF3 (Figure 7K,L). Based on our previous finding that hepatocyte‐released mtDNA promotes macrophage STING activation,^42^ we first determined whether STING activation was caused by mtDNA release. We used DNase I to clear mtDNA and found that DNase I treatment significantly attenuated downstream STING activation in macrophages (Figure S11A–D). Next, we further investigated whether the enhanced STING activation in macrophages was caused by increased release from GPX3‐deficient hepatocytes. We collected the supernatant from primary hepatocytes of GPX3‐deficient mice post‐PH and used it to stimulate WT macrophages (Figure S11E). This stimulation triggered cGAS–STING signalling activation in macrophages (Figure S11G). Concurrently, we detected significantly elevated levels of pro‐inflammatory cytokines in GPX3^fl/fl^Alb^cre^ group, such as IL‐1β, IL‐6 and TNF, but significantly reduced levels of hepatocyte growth factor (HGF)—a key factor promoting hepatocyte proliferation^43^ (Figure S11F).

We next investigated whether these phenotypic changes were caused by GPX3 deficiency‐induced VDAC1 oligomerisation. Using VBIT‐4, we found that hepatocyte mtDNA release was reduced (Figure S11H–J), and VBIT‐4 treatment attenuated STING signalling activation and the release of inflammatory cytokines from macrophages, while increasing HGF release (Figure S11K,L). We further examined the relationship with the antioxidant function of GPX3 and found that NAC could not fully reverse the mtDNA release and STING activation caused by GPX3 deficiency (Figure S12A,B).

To further validate whether GPX3 affects regeneration through this mechanism, we used an adeno‐associated virus (AAV) vector to silence STING. Results showed that compared with GPX3^fl/fl^Alb^cre^ mice, STING silencing rescued the impaired liver regeneration caused by GPX3 deficiency and reduced serum levels of inflammatory cytokines, particularly IFN (Figure S11M‐O). Notably, studies indicate IFN can suppress liver regeneration.^44^ Taken together, these results demonstrate that GPX3 deficiency promotes mtDNA release through VDAC1 oligomerisation, thereby activating the STING signalling pathway and altering macrophage phenotype.

### 3.9 GPX3 overexpression in hepatocytes promotes liver regeneration

**FIGURE 8:**
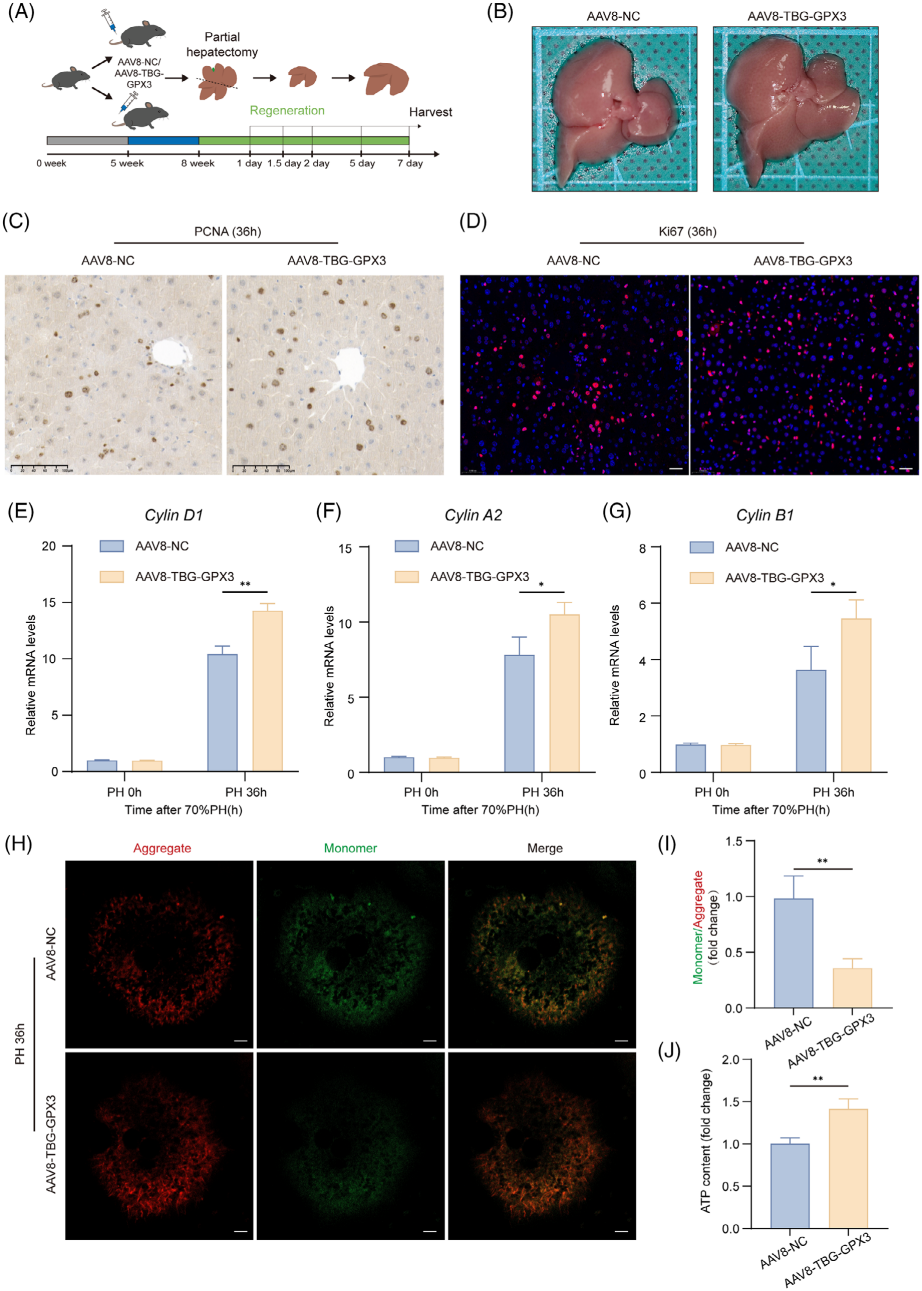
GPX3 overexpression in hepatocytes promotes liver regeneration. (A) Schematic overview of AAV8‐mediated GPX3 overexpression model followed by PH. (B) Liver morphology comparison between AAV8–TBG–GPX3 and AAV8–NC mice 48 h after PH. (C and D) PCNA and Ki67 immunohistochemical staining in AAV8–TBG–GPX3 and AAV8–NC mice at 36 h post‐PH (scale bar = 100 µm). (E–G) RNA levels of proliferation‐related genes (CyclinD1, CyclinA2 and CyclinB1) measured by qRT‐PCR at 36 h after PH, *n* = 5. (H and I) Representative JC‐10‐stained images (H) and quantification of mitochondria depolarisation (I) in primary hepatocytes isolated from AAV8–TBG–GPX3 and AAV8–NC mice at 36 h post‐PH, *n* = 3 (scale bar = 10 µm). (J) ATP content in primary hepatocytes isolated from AAV8–TBG–GPX3 and AAV8–NC mice at 36 h post‐PH, *n* = 3. For experiments involving genotype/treatment and time, statistical significance was determined by two‐way ANOVA followed by Tukey's multiple comparisons test. For single‐time‐point comparisons, an unpaired two‐tailed Student's *t*‐test was used. *p < .05, **p < .01.

Given the protective role of GPX3 in liver regeneration, we investigated its therapeutic potential by delivering an AAV under the control of the liver‐specific thyroxine‐binding globulin (TBG) promoter encoding GPX3 (AAV8–TBG–GPX3) into mice (Figures 8A and S13B). Notably, mice treated with AAV8–TBG–GPX3 exhibited a significantly increased liver‐to‐body weight ratio at 48 h post‐PH compared with control mice (Figures 8B and S13A), suggesting accelerated liver regrowth.

Assessment of hepatocyte proliferation at 36 h post‐PH revealed markedly elevated expression of the proliferation markers Ki67 and PCNA in the GPX3‐overexpressing group (Figure 8C,D), indicating an earlier onset of the regenerative response. Consistently, quantitative PCR analysis demonstrated that Cyclin D1, Cyclin A2 and Cyclin B1 transcripts were significantly up‐regulated in the GPX3‐overexpressing livers compared with controls (Figure 8E–G).

Considering that mitochondrial OXPHOS is impaired following PH (Figure 1C), we next examined mitochondrial function. GPX3 overexpression restored mitochondrial membrane potential, as measured by JC‐10 staining (Figure 8H,I) and significantly enhanced ATP production (Figure 8J). Additionally, mitochondrial ROS generation was markedly reduced in GPX3‐treated mice (Figure S13C,D). Collectively, these findings highlight the therapeutic benefit of GPX3 overexpression in promoting liver regeneration.

### 3.10 GPX3 deficiency in hepatocytes delays liver regeneration following ischemia–reperfusion injury

To investigate the role of GPX3 in hepatic ischemia–reperfusion injury (HIRI) and subsequent tissue repair, we established a mouse model of liver I/R and collected samples at 0, 1, 3, 5 and 7 day post‐reperfusion (Figure S14A). Western blot analysis revealed that hepatocellular GPX3 expression peaked at day 1 post‐reperfusion and progressively declined thereafter (Figure S14B).

Strikingly, hepatocyte‐specific deletion of GPX3 markedly exacerbated liver injury induced by HIRI. Haematoxylin and eosin staining showed that GPX3^fl/fl^Alb^cre^ mice developed more extensive hepatocellular necrosis and karyolysis compared with control GPX3^fl/fl^ mice, with a notably delayed resolution of injury over time (Figure S14C,D). Biochemical analysis further confirmed this observation, as serum AST and ALT levels were significantly elevated in GPX3‐deficient mice and remained higher for a longer duration compared with controls (Figure S14E,F).

To assess the regenerative capacity following injury, we evaluated proliferative markers at day 3 post‐reperfusion. GPX3^fl/fl^Alb^cre^ mice exhibited markedly reduced PCNA positivity and larger necrotic areas relative to control mice (Figure S14G,H), indicating impaired hepatocyte proliferation and delayed tissue regeneration. In addition, GPX3‐deficient mice demonstrated increased expression of pro‐inflammatory cytokines following HIRI, suggesting heightened and prolonged inflammation (Figure S14I–K).

Collectively, these findings demonstrate that hepatocyte‐specific GPX3 plays a critical role in facilitating liver repair after ischemia–reperfusion injury by limiting tissue damage, promoting hepatocyte proliferation and mitigating inflammatory responses. However, unlike in the PH model, the VDAC1–mtDNA–STING axis was not directly examined in the HIRI model in the current study. Therefore, these findings primarily support a protective role of GPX3 at the functional level, while whether the same downstream mechanism operates in HIRI remains to be directly validated in future studies.

## 4 DISCUSSION

Surgical interventions such as PH or liver transplantation remain the predominant curative treatment for both benign and malignant hepatic disorders.^3^, ^45^ Due to the liver volume loss or small size of donor livers, optimal liver regeneration is essential for the recovery of liver function and patient prognosis post‐operation.^46^, ^47^ Hepatocyte proliferation occurs early after PH, during which MQC exerts a critical role by maintaining mitochondrial homeostasis and functions.^48^ However, the underlying regulatory mechanisms remains to be further determined.

Here, we for the first time showed that GPX3 was essential for mitochondrial homeostasis during liver regeneration. Although GPX3 was not the highest‐expressing gene in hepatocytes among all liver cell populations in the public scRNA‐seq dataset, it was inducibly up‐regulated in hepatocytes after PH, suggesting that it may play an important role during liver regeneration. We found that hepatocyte‐specific deletion of GPX3 impaired mitochondrial function and consequently delayed liver regeneration. Mechanistically, GPX3 was able to bind directly to VDAC1 via its A2 domain, leading to down‐regulated VDAC1 oligomerisation and subsequently restored optimal mitochondrial Ca^2^^+^ level and improved MQC. Overexpression of GPX3 improved hepatocyte mitochondrial function and liver regeneration. Although AAV‐mediated GPX3 overexpression promoted liver regeneration in our models, its therapeutic application should be interpreted cautiously, as the optimal timing of intervention, long‐term safety and the potential risk of excessive hepatocyte proliferation remain to be systematically evaluated. These findings suggested that targeting hepatocyte GPX3 or MQC may represent a promising approach to promote liver regeneration.

Mitochondrial function is a key determinant of regenerative capacity.^13^ Mitochondrial dysfunction leads to damage in multiple organs and functions.^49^, ^50^, ^51^ Clinical studies have linked suppressed OXPHOS to poor postoperative liver function and increased risk of liver failure after hepatectomy.^52^, ^53^ Our findings demonstrated that mitochondrial OXPHOS was impaired following PH, coinciding with the study that OXPHOS is suppressed during the early phase of liver regeneration in rats,^16^ which suggested that there may be specific regulatory mechanisms to restore the mitochondria function to provide adequate energy supply during regenerative process. MQC is a critical mechanism to maintain mitochondrial homeostasis by controlling mitochondrial fission, fusion, mitophagy and biogenesis.^17^ MQC has been implicated in liver regeneration by emerging evidence. For example, ATG4B‐mediated mitophagy has been shown to enhance liver regeneration through mesenchymal stem cell‐derived extracellular vesicles.^54^ We previously found that macrophage‐specific deletion of ATG16L1 impairs liver regeneration by disrupting mitophagy.^21^ Mitochondrial fission plays a critical role in liver protection through ALR‐mediated inhibition of Drp1 phosphorylation.^55^ In the current study, we revealed that up‐regulated mitophagy and mitochondria fission but down‐regulated mitochondria fusion and OXPHOS at day 2 after PH, indicating altered mitochondria function and MQC during liver regeneration.

GPX3 is a highly conserved member of the glutathione peroxidase family known for its antioxidant properties and tumour‐suppressive functions in various cancers.^22^, ^56^ Recent studies have also implicated GPX3 in the regulation of mitochondrial function. GPX3 may counteract aging by maintaining mitochondrial homeostasis through potential interactions with UCP1.^24^ GPX3 plays a significant role in mitochondrial redox regulation and protein homeostasis via interaction with Mia40.^23^ Moreover, GPX3 can induce mitochondria‐mediated apoptosis through AMPK/ERK1/2 signalling.^30^ However, its involvement in MQC remained unclear. In our study, GPX3 deficiency led to a marked increase in VDAC1 oligomerisation, thereby disrupting MQC. Mechanistically, GPX3 primarily bond to VDAC1 via its A2 domain to inhibit oligomerisation. It should be noted that in the in vitro experiments to validate the binding site between GPX3 and VDAC1, LPS was used to amplify VDAC1 oligomerisation signals, allowing comparison of the regulatory effects between WT GPX3 and its binding‐deficient mutant. This approach was not intended to mimic the liver regeneration microenvironment, and future studies are needed to develop in vitro models that more closely recapitulate the context of liver regeneration.

VDAC1, a crucial channel protein located on the OMM, plays a pivotal role in preserving MQC.^35^, ^57^, ^58^ VDAC1 dysfunction caused by genetic alterations, oligomerisation, mislocalisation or ubiquitination has been implicated in various diseases.^59^ In the current study, we demonstrated GPX3 deficiency after PH leads to enhanced VDAC1 oligomerisation and subsequent MQC disruption. Notably, treatment with the VDAC1 oligomerisation inhibitor VBIT‐4 restored mitochondrial morphology, respiratory function and regenerative markers, further confirming that GPX3 regulates liver regeneration through modulation of VDAC1 oligomerisation. Notably, given the potential non‐specific effects of VBIT‐4, we further validated our findings using another VDAC1 oligomerisation inhibitor, VBIT‐12 and obtained the same conclusions. Nevertheless, pharmacological inhibitors have inherent limitations, and further genetic epistasis experiments are needed to establish the direct causality between GPX3 and VDAC1. Although the current study has not yet genetically disentangled the enzymatic and structural functions of GPX3 using a catalytically inactive mutant, the NAC treatment results suggest that the protective effect of GPX3 cannot be fully explained by its antioxidant activity alone. In future studies, we plan to further generate and systematically evaluate catalytically inactive GPX3 mutants in liver regeneration models to more clearly define the relative contributions of its enzymatic activity and structure‐dependent function.

Ca^2^^+^ serve as crucial signalling molecules in the regulation of MQC.^34^, ^35^, ^60^, ^61^ VDAC1 constitutes one of the primary pathways for Ca^2^^+^ flux across mitochondrial membranes.^62^ VDAC1 dysfunction caused by oligomerisation may disrupt the equilibrium of Ca^2^^+^ distribution between mitochondrial and cytosolic compartments.^35^, ^57^, ^59^ It has been reported that preventing VDAC1 oligomerisation can maintain mitochondrial Ca^2^^+^ homeostasis and MQC to improve sepsis‐related myocardial injury.^35^ In our study, GPX3 deficiency promoted VDAC1 oligomerisation, resulting in elevated cytosolic Ca^2^^+^ levels coupled with diminished mitochondrial Ca^2^^+^ accumulation. Further investigation revealed changes in MQC. By using VBIT‐4, we found that the abnormal Ca^2^^+^ distribution caused by VDAC1 oligomerisation was restored. Therefore, we speculate that abnormal Ca^2^^+^ distribution is one of the causes of disrupted MQC, although the specific mechanism still needs further validation.

Macrophages constitute one of the largest non‐parenchymal cell populations in the liver. Their interaction with hepatocytes plays a crucial role in regulating liver regeneration.^1^, ^63^ Recent studies indicate that macrophages utilise hepatocyte‐derived glutamate to promote liver regeneration.^18^ mtDNA, a product of mitochondrial dysfunction, can activate the cGAS–STING pathway in downstream macrophages, as demonstrated in our prior work.^41^, ^42^ Furthermore, VDAC1 oligomerisation is known to facilitate mtDNA release.^36^ Therefore, we sought to investigate whether GPX3 deficiency increases mtDNA release by promoting VDAC1 oligomerisation, thereby influencing macrophage phenotype. Our findings demonstrate that GPX3 deficiency enhances mtDNA release from hepatocytes. This increase subsequently activates the cGAS–STING pathway in macrophages, ultimately altering macrophage function. Key changes include reduced secretion of HGF^64^ and increased secretion of pro‐inflammatory factors. Concurrently, STING pathway activation elevates downstream IFN‐a and IFN‐β levels. Collectively, this cascade contributes to delayed liver regeneration. However, the role of macrophages in liver regeneration is dualistic and complex.^65^ While Kupffer cell‐derived IL‐6 is recognised as a key promoter of regeneration,^66^ our study observed an overall suppression of hepatocyte proliferation despite elevated macrophage IL‐6 levels. This discrepancy may arise from differences in experimental timing or the heterogeneous nature of macrophage populations not further analysed here. The underlying mechanisms require further elucidation.

## AUTHOR CONTRIBUTIONS

X. W., H. Z. and Z. R. designed the experiments. Y. W., J. X., Z. Z. and Y. Z. performed the experiments. H. H. and Y. G. performed the data analyses. Y. W., J. X. and F. Y. wrote the manuscript. Y. T., F. Y., S. Z. and C. F. provided technical support. X. W. and H. Z. organised and supervised the study.

## CONFLICT OF INTEREST STATEMENT

The authors declare no conflicts of interest.

## ETHICS STATEMENT

All animals were treated humanely, and all animal procedures met the relevant legal and ethical requirements according to the protocols (number IACUC‐2311005) approved by the Institutional Animal Care and Use Committee of Nanjing Medical University.

## CONSENT

The authors have nothing to report.

## CLINICAL TRIAL NUMBER

With ethical approval (Approval No. 2023‐SRFA‐377), liver tissue specimens were obtained from three patients who underwent PVE at the First Affiliated Hospital of Nanjing Medical University. Both normal liver tissues (away from the embolised area) and regenerating liver tissues (from the hypertrophied lobe after PVE) were collected from each patient. None of the patients had received prior anti‐tumour treatment, and written informed consent was obtained from each individual.

## Supporting information



SUPPORTING INFORMATION

SUPPORTING INFORMATION

## Data Availability

Any reasonable requests for access to available data underlying the results reported in this article will be considered. Such proposals should be submitted to the corresponding author.
