# Supplementary material for: Glutathione peroxidase 3 preserves hepatocyte mitochondrial quality control to enhance macrophage pro‐regenerative phenotype during liver regeneration
Source: Clin Transl Med. 2026 May 29;16(6):e70695. doi: 10.1002/ctm2.70695 (PMC13240042; doi:10.1002/ctm2.70695)
Supplement: Supplementary file 1 — SUPPORTING INFORMATION [file CTM2-16-e70695-s001.docx]

Supporting Information

**Glutathione peroxidase 3 preserves hepatocyte mitochondrial quality control to enhance macrophage pro-regenerative phenotype during liver regeneration**

Yuechen Wang^1#^, Jian Xu^1#^, Zeyu Zhu^1#^, Ye Zhang^1 #^, Haoran Hu^1^, Yiyun Gao^1^, Yuting Tao^1^, Feifan Yao^1^, Suiqing Zhou^1^, Weizhe Zhong^1^, Zhuqing Rao^2*^, Haoming Zhou^1*^, Xuehao Wang^1,3*^

1. Hepatobiliary Center, The First Affiliated Hospital with Nanjing Medical University, Key Laboratory of Liver Transplantation, Chinese Academy of Medical Sciences, Key Laboratory of Hepatobiliary Tumors, National Health Commission, Jiangsu Provincial Medical Innovation Center, Jiangsu Provincial Medical Key Laboratory, Nanjing, Jiangsu Province, China.
2. Department of Anesthesiology and Perioperative Medicine, The First Affiliated Hospital with Nanjing Medical University, Nanjing 210029, China.
3. Collaborative Innovation Center for Cancer Personalized Medicine, Nanjing Medical University, Nanjing, Jiangsu Province, China.

^#^ These authors contributed equally to this work.

** Corresponding author. Xuehao Wang, Hepatobiliary/Liver Transplantation Center, First Affiliated Hospital with Nanjing Medical University, Nanjing, China, 210029. Email: wangxh@njmu.edu.cn.

* Corresponding author. Haoming Zhou, Hepatobiliary/Liver Transplantation Center, First Affiliated Hospital with Nanjing Medical University, Nanjing, China, 210029. Email: [hmzhou@njmu.edu.cn](mailto:hmzhou@njmu.edu.cn).

* Corresponding author. Zhuqing Rao, Department of Anesthesiology, The First Affiliated Hospital with Nanjing Medical University, Nanjing, China, 210029.

Email: [zhuqingrao@njmu.edu.cn](mailto:zhuqingrao@njmu.edu.cn)

**Table of contents:**

Materials and methods...............................................................................2

Supplementary figures..................................................................................19

Supplementary tables.........................................................................37

Supplementary references.........................................................................39

**Materials and methods**

**Animal studies**

The wild-type (WT), GPX3-FloxP (GPX3^fl/fl^), Alb-Cre mice used in this study were obtained from GemPharmatech Co (Nanjing, Jiangsu, China). Hepatocyte-specific GPX3 knockout (HKO) mice were generated by breeding GPX3-FloxP with Alb-Cre mice. Briefly, homozygous GPX3^fl/fl^ mice were first bred with homozygous Alb-Cre mice, and the heterozygous offspring (for both GPX3 and Cre) were back-crossed with homozygous GPX3^fl/fl^ mice. This backcrossing strategy was used to generate GPX3^fl/fl^Alb^Cre^ mice together with GPX3^fl/fl^ littermate controls on a comparable genetic background.

All mice were kept under specific pathogen‑free conditions on a 12h light/dark cycle, with free access to water and standard chow supplemented as required. Humane treatment of animals was ensured throughout, and all procedures were conducted in compliance with the relevant legal and ethical standards, following protocol number IACUC‑2311005 approved by the Institutional Animal Care and Use Committee of Nanjing Medical University.

**Clinical specimens**

Liver tissue specimens were obtained from three patients who underwent portal vein embolization (PVE). Both normal liver tissues (away from the embolized area) and regenerating liver tissues (from the hypertrophied lobe after PVE) were collected. Freshly resected tissue samples were immediately frozen in liquid nitrogen or fixed in 4% paraformaldehyde. Histological and pathological examinations and grading were performed by two experienced pathologists. Written informed consent was obtained from each patient prior to treatment, and the study protocol was approved by the Ethics Committee of the First Affiliated Hospital of Nanjing Medical University.

**Adeno-associated virus (AAV)**

To achieve hepatocyte-specific overexpression of glutathione peroxidase 3 (GPX3), an adeno-associated virus (AAV) vector carrying the mouse GPX3 coding sequence under the control of the liver-specific thyroxine-binding globulin (TBG) promoter was constructed (AAV8-TBG-GPX3). To silence STING in macrophage, AAV-Tmem173 was constructed. The Adenoassociated viruses generated were then amplified at the Genechem Co., Ltd. (Shanghai, China). Male C57BL/6 mice (6 weeks old) received a single tail vein injection of 100 μL AAV8-TBG-GPX3 or AAV-Tmem173 viral suspension (viral titer > 5×10^11^ vg/mL).

To verify the efficiency of GPX3 overexpression mediated by AAV8-TBG-GPX3, mouse livers were harvested at two weeks after tail vein injection of AAV8-TBG-GPX3 (or control virus). Primary hepatocytes were isolated using a two-step collagenase perfusion method. Total protein was extracted from freshly isolated primary hepatocytes, and GPX3 protein expression levels were detected by Western blot. GAPDH was used as a loading control. GPX3 expression levels were compared between the AAV8-TBG-GPX3 group and the control group (injected with empty vector) to confirm the overexpression efficiency.

**Generation of mouse hepatectomy model**

A mouse partial hepatectomy model was established to study liver regeneration. Briefly, male C57BL/6 mice (8–10 weeks old) were anesthetized with isoflurane, and a 70% partial hepatectomy was performed according to the standard method as described.(1) The left lateral and median lobes of the liver were carefully ligated and surgically removed under sterile conditions. Sham-operated mice underwent the same surgical procedure without liver lobe resection. After surgery, mice were placed on a heating pad and monitored until full recovery. Liver tissues were collected at the indicated time points for further analysis.

To inhibit VDAC1 oligomerization, VBIT-4 (TargetMol, China) was dissolved in DMSO to prepare a 20 mg/mL stock solution. Before administration, 100 μL of the DMSO stock solution was mixed sequentially with 400 μL PEG300 (TargetMol, China), 50 μL Tween 80 (TargetMol, China), and 450 μL PBS, with thorough mixing until clear, to obtain a 2 mg/mL working solution. Mice were injected intraperitoneally at a dose of 20 mg/kg body weight. For a mouse weighing approximately 25 g, the injection volume was approximately 250 μL. The injection was started one day before partial hepatectomy and repeated once daily after surgery until sacrifice. Control mice received an equal volume of vehicle (the same mixture of DMSO/PEG300/Tween 80/PBS at the same ratio).

We also used VBIT-12 (Selleck, China). VBIT-12 was dissolved in DMSO to prepare a 20 mg/mL stock solution. Before administration, 50 μL of the DMSO stock solution was sequentially mixed with 400 μL PEG300, 50 μL Tween 80, and 500 μL ddH₂O, with thorough mixing until clear, to obtain a 2 mg/mL working solution. Mice were intraperitoneally injected with VBIT-12 at a dose of 20 mg/kg body weight. For a mouse weighing approximately 25 g, the injection volume was approximately 250 μL. The injection was started one day before partial hepatectomy and repeated once daily after surgery until sacrifice. Control mice received an equal volume of vehicle (the same mixture of DMSO/PEG300/Tween 80/ddH₂O at the same ratio).

To eliminate ROS, NAC (TargetMol, China) was dissolved in sterile PBS to prepare a 150 mg/mL stock solution. Before administration, 100 μL of the PBS stock solution was mixed sequentially with 400 μL PBS, 50 μL PBS, and 450 μL PBS, with thorough mixing until clear, to obtain a 15 mg/mL working solution. Mice were injected intraperitoneally at a dose of 150 mg/kg body weight. For a mouse weighing approximately 25 g, the injection volume was approximately 250 μL. The injection was started two days before partial hepatectomy and repeated once daily after surgery until sacrifice. Control mice received an equal volume of vehicle (sterile PBS).

**Mouse liver IRI model**

A partial hepatic warm ischemia-reperfusion injury (IRI) model was established in mice as described.(2) After successful anaesthesia with 2.5% isoflurane, heparin (100 mg/kg) was injected into the mice. Following midline laparotomy, a non-traumatic microvascular clamp was applied to interrupt the portal vein and hepatic artery blood supply to the left and median liver lobes, inducing segmental ischemia. After 90 minutes of ischemia, the clamp was removed to initiate reperfusion. Throughout the procedure, mice were maintained under anesthesia and placed on a warming pad to preserve body temperature. Liver tissues were harvested at 0, 12, 24, 72, 120, and 168 hours post-reperfusion. Sham-operated mice underwent the same surgical procedure without vascular occlusion and served as controls.

**Analysis of single-cell RNA sequencing data**

Publicly available scRNA-seq datasets (CNP0002310) were obtained from the CNGBdb (China National GeneBank DataBase).(3) Raw data were processed and analyzed using the Seurat R package (v4.3.0) following standard workflows for quality control, normalization, and dimensionality reduction. Cell clusters were identified via tSNE and UMAP, with marker genes determined by differential expression analysis (Wilcoxon rank-sum test, adjusted *p* < 0.05).

**Cell lines and cell culture**

The AML12 cell line and NIH/3T3 were obtained from the Shanghai Institutes for Biological Sciences, Chinese Academy of Sciences (Shanghai, China). All cell lines were cultured in DMEM (GIBCO, NY, USA) supplemented with 10% fetal bovine serum (GIBCO) and 1% penicillin-streptomycin (GIBCO). All cell lines were maintained in a 5% CO2 humidified incubator (Thermo Fisher Scientific, MA, USA) at 37°C.

**Chromatin immunoprecipitation (ChIP) assay**

ChIP-qPCR was performed using a ChIP Assay Kit (Beyotime, Shanghai, China) according to the manufacturer’s instructions. Briefly, cells or liver tissues were crosslinked with 1% formaldehyde for 10 min at room temperature, and the reaction was quenched with 125 mM glycine for 5 min. Samples were then washed with cold PBS and lysed. Chromatin was fragmented by sonication to an average size of 200–500 bp. The sheared chromatin was incubated overnight at 4 °C with an anti-HIF-1a antibody or normal rabbit IgG as a negative control. Immune complexes were captured with protein A/G magnetic beads, washed sequentially, and eluted. Crosslinks were reversed, and DNA was purified for qPCR analysis. qPCR was performed using primers flanking the predicted HIF-1α-binding site in the GPX3 promoter. Enrichment was calculated relative to input chromatin and normalized to the IgG control.

**Isolation of mouse primary hepatocytes and macrophages**

Livers were perfused in situ via the portal vein with calcium- and magnesium-free Hank’s balanced salt solution (HBSS) supplemented with 2% heat-inactivated fetal bovine serum (FBS), followed by perfusion with 0.27% collagenase IV (Sigma-Aldrich, St. Louis, MO, USA). The digested livers were excised, minced, and filtered through 70-μm nylon mesh cell strainers (BD Biosciences, San Diego, CA, USA) to obtain single-cell suspensions. Liver cells were resuspended in DMEM containing 10% FBS and fractionated into hepatocytes and nonparenchymal cells (NPCs), including Kupffer cells (KCs), as follows.

Cells were first centrifuged at 50 × g for 2 minutes to pellet hepatocytes. The supernatant was collected and centrifuged three times at 50 × g to maximize hepatocyte recovery. Pelleted hepatocytes were resuspended in 40% cold Percoll solution (P1644, Sigma-Aldrich) and centrifuged at 150 × g for 7 minutes to enrich viable hepatocytes. Cells were washed once with DMEM containing 10% FBS and then resuspended in Williams’ E medium supplemented with a hepatocyte thawing and plating supplement pack. Cells were plated on collagen type I-coated plates and incubated for 3 hours. For overnight culture or longer, maintenance was performed in Williams’ E medium with a hepatocyte maintenance supplement pack.

**DNase I treatment**
To assess the contribution of extracellular mtDNA during liver regeneration, mice were administered DNase I (TargetMol, China) by intraperitoneal injection at a dose of 10 U/100 μL per mouse beginning after partial hepatectomy (PH) and continued once daily until sacrifice. Control mice received an equal volume of sterile saline. Samples were collected at the indicated time points for analysis of extracellular mtDNA levels, macrophage activation, and liver regeneration-related phenotypes.

**Protein isolation and western blotting (WB)**

Cells and liver tissue samples were lysed using RIPA lysis buffer (Beyotime, Shanghai, China), and protein concentrations were determined with the Enhanced BCA Protein Assay Kit (Beyotime). Equal amounts of protein were separated by SDS-PAGE using 10% or 12.5% polyacrylamide gels and transferred onto polyvinylidene difluoride (PVDF) membranes (Millipore, Billerica, MA, USA). Membranes were blocked at room temperature for 2 hours using blocking buffer (Beyotime), followed by overnight incubation at 4°C with primary antibodies. After three 15-minute washes with TBST (Tris-buffered saline with 0.1% Tween-20), membranes were incubated with HRP-conjugated secondary antibodies for 1 hour at room temperature. After additional washes, signals were developed using an enhanced chemiluminescence (ECL) detection kit (UElandy, Suzhou, China) and visualized with a chemiluminescence imaging system (Vilber, Paris, France). Band intensities were quantified using ImageJ software (NIH, Bethesda, MD, USA). Details of the antibodies used are listed in Supplementary Table S1.

**VDAC1 cross-linking assay**

The VDAC1 cross-linking assay was performed as previously reported.(4, 5) Cells were washed twice with cold PBS (pH 8.0) and centrifuged (3,000 × g, 5 min, 4 °C). Pellets were resuspended in 740 μL of 0.5 mM EGS (Thermo Fisher Scientific) in PBS (pH 8.0) and crosslinked for 30 min at RT. The reaction was quenched with 10 μL of 1.5 M Tris-HCl (pH 7.8) for 15 min. Cells were lysed, and protein concentration was determined by BCA assay. Equal amounts (50 μg) were resolved by 7.5% SDS-PAGE and immunoblotted with anti-VDAC1 antibody. It should be noted that VDAC1 oligomerization in this study was not assessed directly under non-denaturing electrophoretic conditions, but was detected by EGS chemical cross-linking followed by SDS-PAGE and immunoblotting. Therefore, the observed dimeric, trimeric, and higher-order VDAC1 bands represent EGS-stabilized oligomeric forms.

**RNA isolation, reverse transcription, and quantitative PCR (qPCR)**

Total RNA was isolated from liver tissues and cell lines using the Total RNA Isolation Kit V2 (Vazyme, Nanjing, China) according to the manufacturer’s instructions. Complementary DNA (cDNA) was synthesized using the PrimeScript RT Reagent Kit (Vazyme). Quantitative real-time PCR (qRT-PCR) was performed using SYBR Green Master Mix (Vazyme) on an Applied Biosystems 7900HT Fast Real-Time PCR System (Thermo Fisher Scientific, MA, USA). Relative mRNA expression levels were calculated using the 2^^−ΔΔCt^ method, with β-actin used as the internal control. The primers used for this analysis are listed in Supplementary Table S2.

**Immunofluorescence assay**

For immunofluorescence analysis, cells were seeded onto confocal culture dishes on day 1. On the following day, cells were fixed with 4% paraformaldehyde at room temperature for 10 minutes and then blocked with blocking buffer (Beyotime, Shanghai, China) for 2 hours at room temperature. Cells were incubated overnight at 4°C with primary antibodies. On day 3, cells were washed three times with washing buffer (Beyotime) at room temperature. Subsequently, cells were incubated with fluorescent secondary antibodies (1:200 dilution) for 2 hours at room temperature in the dark. After another three washes, nuclei were counterstained with DAPI (Beyotime). Samples were stored at 4°C in the dark until imaging. Images were acquired using an Olympus Fluoview 1200 confocal microscope and processed with ImageJ software. Details of antibodies used are provided in Supplementary Table S1.

**Immunohistochemistry and hematoxylin and eosin staining**

Liver tissues were fixed in 4% paraformaldehyde, embedded in paraffin, and sectioned at 4 μm thickness. For hematoxylin and eosin (H&E) staining, sections were processed using standard protocols to assess histological changes. For immunohistochemistry (IHC), sections were deparaffinized, rehydrated, and subjected to antigen retrieval. Endogenous peroxidase activity was blocked, followed by incubation with primary antibodies at 4°C overnight. After washing, sections were incubated with HRP-conjugated secondary antibodies and visualized using DAB substrate. Nuclei were counterstained with hematoxylin. Image analysis was performed using ImageJ software.

**Hepatocellular function assay**

Blood samples were centrifuged to obtain serum, and the levels of aspartate aminotransferase (ALT) and alanine aminotransferase (AST) were measured using an automatic chemical analyzer (Olympus Company, Tokyo, Japan).

**Reactive Oxygen Species (ROS) Detection**

Intracellular reactive oxygen species (ROS) levels were measured using the ROS Assay Kit (Beyotime, Shanghai, China) following the manufacturer’s instructions. Briefly, cells were incubated with 10 μM DCFH-DA, a fluorescent ROS-sensitive probe, at 37°C for 30 minutes in the dark. After incubation, cells were washed three times with serum-free medium to remove excess dye. Fluorescence was observed using a fluorescence microscope (Zeiss, Oberkochen, Germany), and image analysis was performed using ImageJ software.3

**Oxygen Consumption Rate (OCR) Measurement**

The mitochondrial oxygen consumption rate (OCR) was measured using the Seahorse XF Cell Mito Stress Test Kit on a Seahorse XFe96 Extracellular Flux Analyzer (Agilent Technologies, Santa Clara, CA, USA) according to the manufacturer’s protocol. Cells were seeded into XF96 cell culture microplates and incubated overnight. Prior to the assay, cells were equilibrated in Seahorse XF assay medium (supplemented with 10 mM glucose, 2 mM glutamine, and 1 mM pyruvate) at 37°C in a non-CO₂ incubator for 1 hour. OCR was measured at baseline and following sequential injections of oligomycin (1 μM), FCCP (1 μM), and a mixture of rotenone and antimycin A (0.5 μM each). Data were normalized to cell number or protein content and analyzed using Wave software (Agilent).

Primary mouse hepatocytes were seeded into Seahorse XF24 cell culture plates at a density of 2 × 10⁴ cells per well and cultured overnight in DMEM containing 10% FBS. Before the assay, the culture medium was replaced with Seahorse assay medium (supplemented with 10 mM glucose, 2 mM glutamine, and 1 mM pyruvate). The oxygen consumption rate (OCR) was continuously monitored using a Seahorse XF24 Extracellular Flux Analyzer (Agilent Technologies, Santa Clara, CA, USA). Parameters including basal respiration, ATP-linked respiration, maximal respiration, and non‑mitochondrial respiration were measured sequentially. Each experimental condition was performed with 3–5 replicate wells, and the experiment was repeated at least three times.

**Mitochondrial Membrane Potential Assay**

Mitochondrial membrane potential (ΔΨm) was assessed using the JC-10 Mitochondrial Membrane Potential Assay Kit (Yeasen, Shanghai, China) following the manufacturer’s protocol. Briefly, cells were incubated with JC-10 working solution at 37°C for 30 minutes in the dark. After incubation, cells were washed gently with assay buffer and imaged using a fluorescence microscope (Zeiss, Oberkochen, Germany) equipped with ZEN software. JC-10 monomers (indicative of depolarized mitochondria) emit green fluorescence, while aggregates (polarized mitochondria) emit red fluorescence. The ratio of red to green fluorescence was analyzed using ImageJ software to evaluate changes in ΔΨm.

**Mitochondria and cytosolic dsDNA measurements**

The PicoGreen dsDNA Reagent and Kit (Invitrogen, no. P11496) and MitoTracker Red CMXRos (Invitrogen, no. M5712) were used at 0.3% v/v and 100 nM in serum-free DMEM for 30 min at 37 °C to co-localize dsDNA and mitochondria.

**Mitophagy analysis using Lv-mtKeima-COX8**Mitophagy in primary hepatocytes was assessed using the Lv-mtKeima-COX8 reporter system (Hanbio, Shanghai, China). Briefly, primary hepatocytes were seeded onto glass-bottom dishes and infected with Lv-mtKeima-COX8 at the indicated multiplicity of infection (MOI) according to the manufacturer’s instructions. After 24–48 h of infection, cells were washed and maintained in fresh culture medium before imaging. mtKeima fluorescence was detected using a Stellaris STED confocal microscope system (Leica Microsystems, Wetzlar, Germany). Signals from the neutral mitochondrial compartment and the acidic lysosomal compartment were recorded separately, and the ratio of acidic to neutral mtKeima fluorescence was used as an indicator of mitophagy. Fluorescence intensity was quantified using ImageJ in individual cells or predefined regions of interest (ROIs). All imaging parameters were kept constant within each experiment.

**ATP Assay**

Intracellular ATP levels were measured using the ATP Assay Kit (Beyotime, Shanghai, China) following the manufacturer’s protocol. Briefly, cells or tissue lysates were prepared using the lysis buffer provided in the kit. After centrifugation, supernatants were collected and mixed with the luciferase-based detection solution. Luminescence intensity was immediately measured using a microplate luminometer (Thermo Fisher Scientific, MA, USA). ATP concentrations were calculated from a standard curve and normalized to total protein content. All samples were analyzed in triplicate

**Transmission Electron Microscopy (TEM)**

Liver tissue samples were fixed in 2.5% glutaraldehyde in 0.1 M phosphate buffer (pH 7.4) at 4°C overnight, followed by post-fixation in 1% osmium tetroxide for 1 hour at room temperature. After dehydration through a graded ethanol series and embedding in epoxy resin, ultrathin sections (approximately 70 nm) were cut using an ultramicrotome and stained with uranyl acetate and lead citrate. Sections were examined using a transmission electron microscope (JEM-1400FLASH, JEOL Ltd., Tokyo, Japan). Representative images were acquired digitally.

**Mitochondrial Staining**

Mitochondria were labeled using MitoTracker® Red CMXRos (Yeasen, Shanghai, China) according to the manufacturer’s instructions. Briefly, cells were incubated with 100 nM MitoTracker Red CMXRos diluted in serum-free medium at 37°C for 30 minutes in the dark. After staining, cells were washed three times with PBS to remove excess dye. Fluorescence images were acquired using a Stellaris STED confocal microscope system (Leica Microsystems, Wetzlar, Germany). Mitochondrial morphology and fluorescence intensity were analyzed using ImageJ software.

**Co-immunoprecipitation (Co-IP) assay**

Co-immunoprecipitation (Co-IP) was performed to assess protein–protein interactions. Cells were lysed in IP lysis buffer (containing protease and phosphatase inhibitors) on ice for 30 minutes. After centrifugation at 12,000 × g for 15 minutes at 4°C, the supernatants were collected and precleared with protein A/G agarose beads (Santa Cruz Biotechnology) for 1 hour at 4°C. The cleared lysates were then incubated overnight at 4°C with specific primary antibodies or control IgG, followed by incubation with fresh protein A/G agarose beads for an additional 2–4 hours. After extensive washing with lysis buffer, the immunoprecipitates were eluted by boiling in SDS sample buffer and analyzed by SDS-PAGE and western blotting. Input and IP samples were probed with the indicated antibodies.

**Immunoprecipitation coupled with mass spectrometry (IP/MS)**

The IP/MS procedure was performed as previously described.(6) Briefly, proteins were extracted from transfected cells using lysis buffer, and immunoprecipitation was carried out with specific primary antibodies and Protein A/G agarose beads (Santa Cruz Biotechnology, Santa Cruz, CA, USA). Bound proteins were eluted from the beads using SDT lysis buffer, followed by boiling at 100°C for 3 minutes and ultrasonic disruption. After centrifugation at 16,000 × g for 15 minutes, the supernatant containing solubilized proteins was collected. Protein digestion was performed using the filter-aided sample preparation (FASP) method established by Wisniewski and Zougman.(7) Briefly, detergent, dithiothreitol (DTT), and iodoacetamide were sequentially added in a urea–ammonium bicarbonate buffer to reduce and alkylate cysteine residues. Proteins were then digested with 2 μg of trypsin (Promega, Madison, WI, USA) at 37°C overnight. The resulting peptides were desalted and purified using C18 StageTips, and subjected to LC-MS/MS analysis using a Q Exactive Plus mass spectrometer coupled with an Easy-nLC 1200 system (Thermo Fisher Scientific, Waltham, MA, USA). Raw data were processed using MaxQuant software (version 1.6.1.0; Max Planck Institute of Biochemistry, Martinsried, Germany).

**Flow cytometry analysis**

Hepatic immune cells were harvested from the livers of mice subjected to partial hepatectomy (PHx) and enriched via density gradient centrifugation using 25% and 50% Percoll (Cytiva, 17089101) at 800 × g. Cells were first stained with a fixable viability dye (eBioscience), then blocked with Fc receptor antibody (BD Biosciences) and labeled with the following surface markers (Biolegend) for 30 min: CD45, F4/80, Ly6G, Ly6C, CD11b, CD3, CD4, CD8, and NK1.1. Flow cytometric analysis was performed on a Beckman system, and data were analyzed using FlowJo software (Treestar). For sorting of hepatic macrophages, the same staining panel was applied, and target cells were isolated on a Beckman Coulter sorter with a final purity exceeding 90%. All acquisition and processing were conducted with FlowJo.

**Cytosolic Ca²⁺ imaging**

Intracellular cytosolic Ca²⁺ levels were assessed using the Fluo-8 AM calcium indicator (Abcam, Cambridge, UK) according to the manufacturer’s instructions. Briefly, cells were incubated with 5 μM Fluo-8 AM diluted in serum-free medium containing 0.02% Pluronic F-127 at 37 °C for 30 min in the dark. After incubation, cells were washed with Hank’s balanced salt solution (HBSS) to remove excess dye and equilibrated for an additional 10 min at 37 °C. Fluorescence signals were detected using a Stellaris STED confocal microscope (Leica Microsystems, Wetzlar, Germany).

**Mitochondrial Ca²⁺ imaging and quantification**

Mitochondrial Ca²⁺ was evaluated using Rhod-2 AM under conditions optimized for mitochondrial accumulation. After dye loading, cells were washed and allowed to undergo de-esterification before imaging. For analysis, Rhod-2 signals with punctate/perinuclear distribution consistent with mitochondrial localization were preferentially quantified. All images were acquired using identical microscope settings within each experiment. Fluorescence intensity was analyzed using ImageJ after background subtraction and quantified in individual cells or predefined regions of interest (ROIs). Values were normalized to the corresponding control or baseline values and are presented as relative fluorescence intensity. No in situ calibration was performed; therefore, the data reflect relative changes in cytosolic or mitochondrial Ca²⁺ rather than absolute Ca²⁺ concentrations.

**Enzyme-linked immunosorbIMent assay (ELISA)**

The levels of IL-1β, TNF-α, IL-6, IFN-α, IFN-ꞵ, and HGF in serum or cell supernatants were detected using ELISA kits (Thermo Fisher Scientific) according to the manufacturer's instructions.

**Statistical analysis**

All data were analyzed using SPSS Statistics (version 19.0; SPSS, NY, USA) and GraphPad Prism 8.0 (GraphPad Software, CA, USA). Results are presented as mean ± SD or mean ± SEM from at least three independent experiments, as indicated in the figure legends. Normality was assessed using the Shapiro–Wilk test. For comparisons between two groups, a two-tailed unpaired Student’s *t* test was used for normally distributed data, whereas the Mann–Whitney test was used for non-normally distributed data. For experiments involving a single factor with more than two groups, one-way ANOVA followed by Tukey’s multiple comparisons test was applied. For experiments involving two factors, such as genotype/treatment and time, two-way ANOVA followed by Tukey’s multiple comparisons test was used to assess the main effects and their interaction. Welch’s ANOVA with Dunnett’s T3 post hoc test was used when appropriate. The specific statistical methods used for each experiment are indicated in the corresponding figure legends. Statistical significance was considered at p < 0.05 (n.s. = not significant, *p < 0.05, **p < 0.01, and ***p < 0.001).

**Supplementary Figure**

**Figure S1**

**
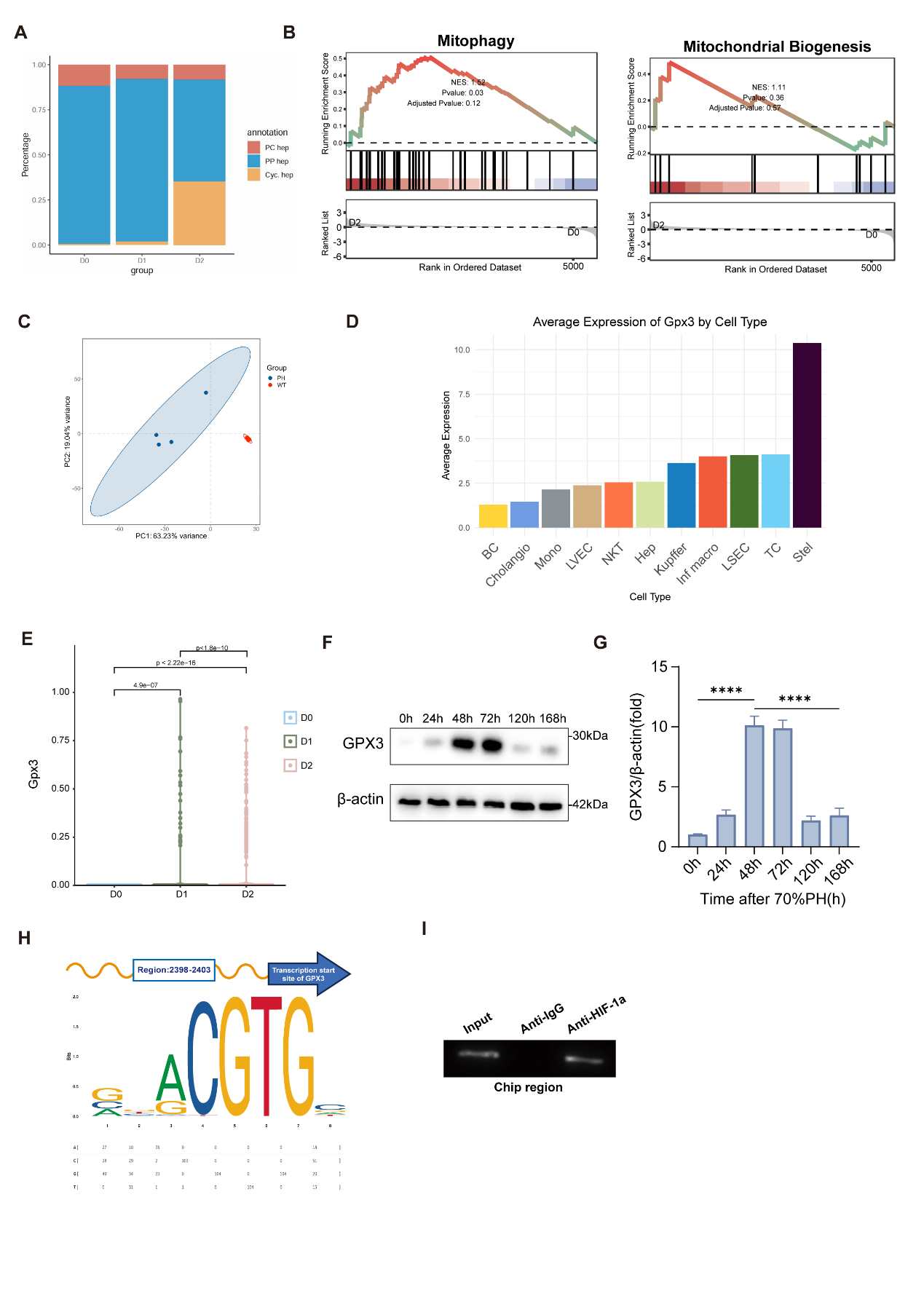
 Figure S1. GPX3 expression is upregulated following PH.** （A）Relative proportions of hepatocyte subclusters, including periportal hepatocytes (PP hep), pericentral hepatocytes (PC hep), and cycling hepatocytes (Cyc. hep), at D0, D1, and D2 after PH. (B) GSEA for mitophagy and mitochondrial biogenesis in hepatocytes during liver regeneration. (C) Principal component analysis (PCA) of transcriptomic profiles between PH and control groups, n=4/group. (D) Average GPX3 expression across major liver cell types in the public scRNA-seq dataset. (E) Percentage of hepatocyte subclusters at 0, 24, and 48h post-PH. (F, G) Western blot analysis and quantitation of GPX3 in liver tissues at indicated time points post-PH, n=3. (H) Predicted HIF-1α-binding motif in the GPX3 promoter identified by JASPAR. (I) Putative HIF-1a-binding sites identified in the promoter region of GPX3 gene. For experiments involving genotype/treatment and time, statistical significance was determined by two-way ANOVA followed by Tukey’s multiple comparisons test. ****p <0.001

**Figure S2**

**
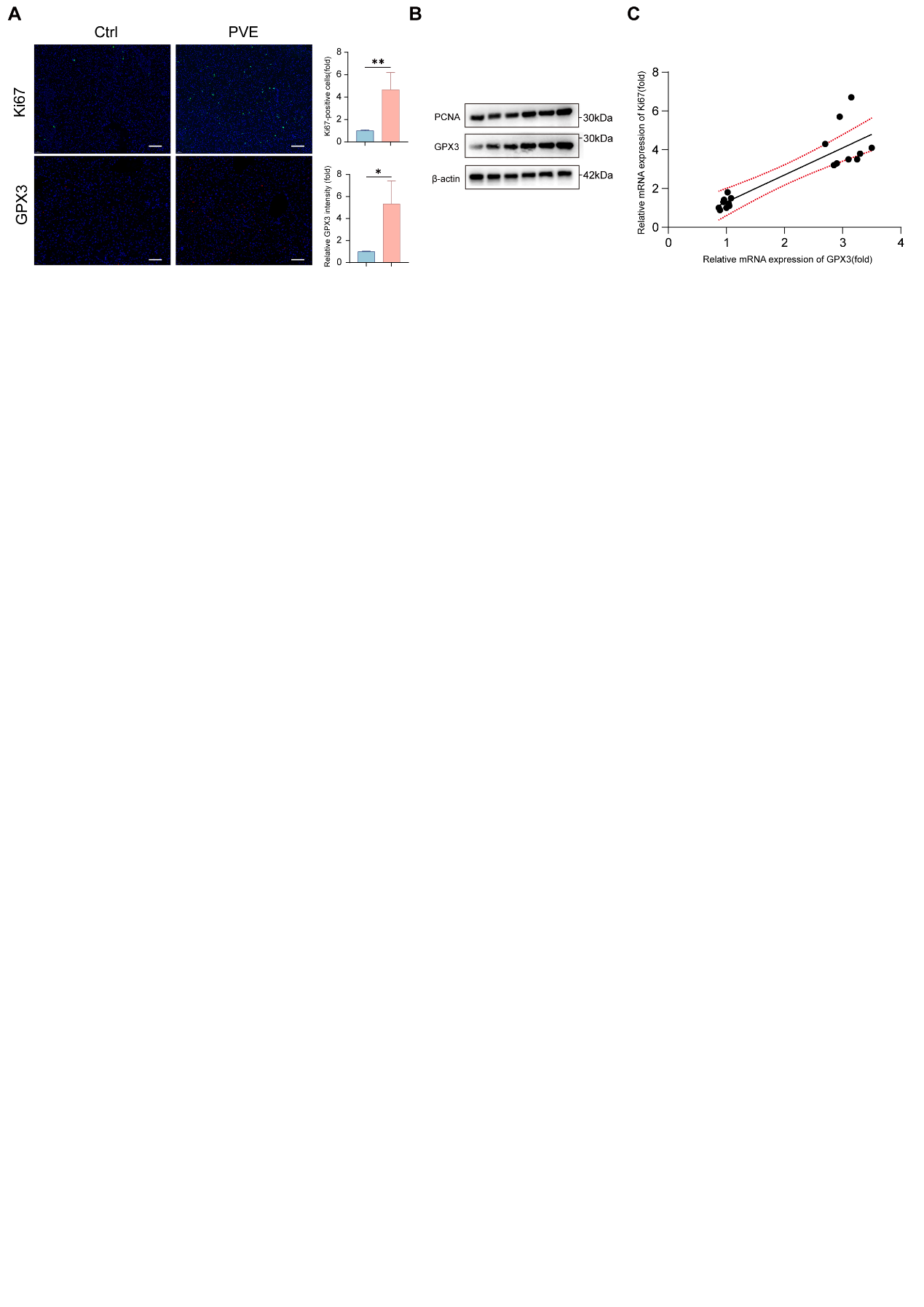
**

**Figure S2. GPX3 is upregulated in human regenerating liver tissues.**

(A) Representative immunofluorescence staining of Ki67 and GPX3 in liver tissues from the control (Ctrl) and portal vein embolization (PVE) groups (scale bar=50μm). Quantification of Ki67-positive cells and relative GPX3 fluorescence intensity is shown on the right, n=3. (B) Western blot analysis of PCNA and GPX3 protein expression in liver tissues, n=3. (C) Correlation analysis between GPX3 and Ki67 mRNA expression levels in liver samples. Statistical significance was determined by an unpaired two-tailed Student’s t-test or one-way ANOVA followed by Tukey’s multiple comparisons test, as appropriate. *p <0.05, **p <0.01

**Figure S3**


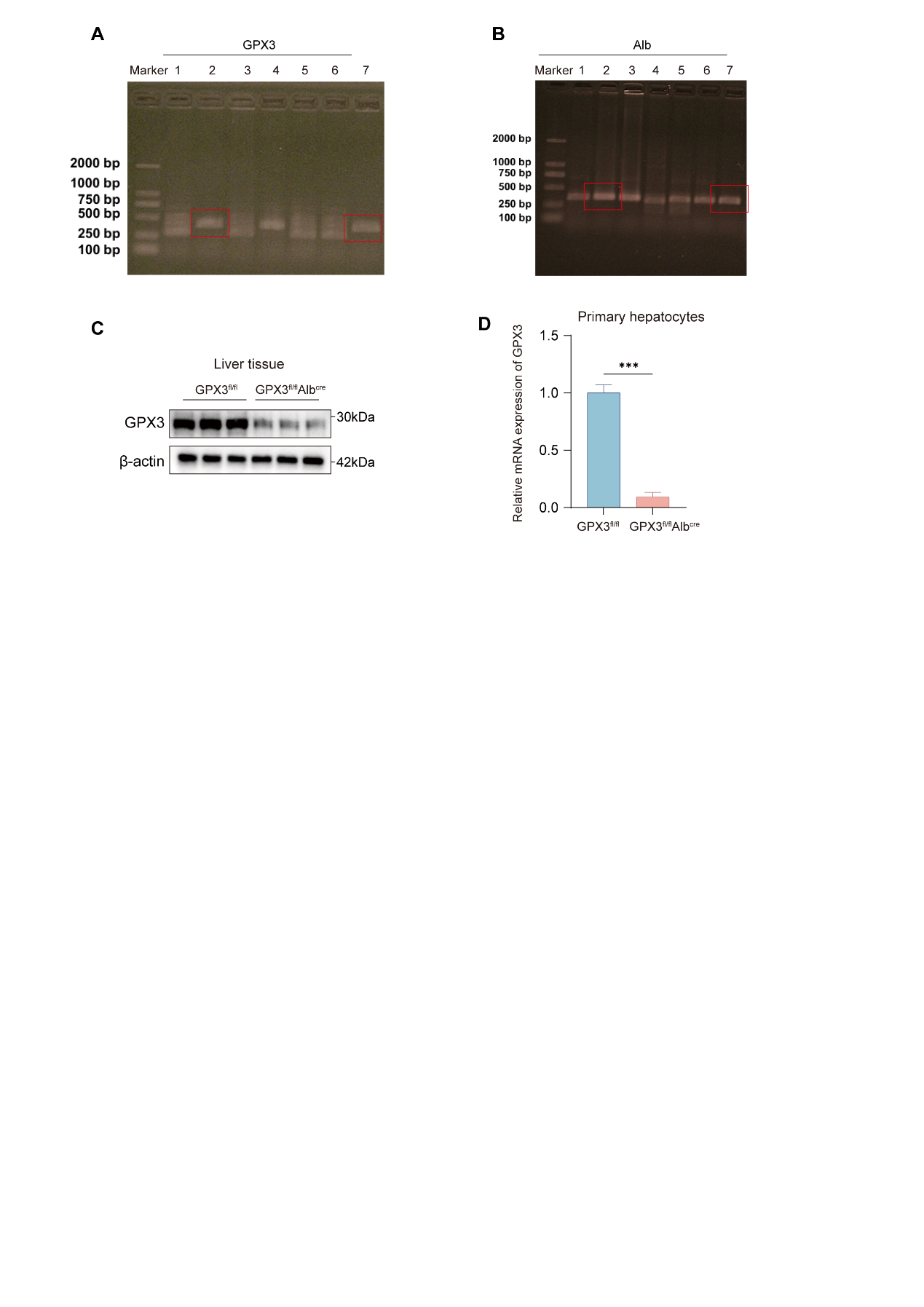


**Figure S3. GPX3 expression is upregulated following PH.** (A) PCR genotyping of the GPX3 floxed allele. (B) PCR genotyping of Alb-Cre. Samples highlighted by red boxes indicate mice with the GPX3^fl/fl^ and Alb^cre^ used as hepatocyte-specific knockout mice; in this experiment, lanes 2 and 7 represent the knockout genotype. (C) Western blot analysis of GPX3 protein expression in liver tissues from GPX3^fl/fl^Alb^cre^ and GPX3^fl/fl^ mice, n=3. (D) qPCR analysis of GPX3 mRNA expression in primary hepatocytes isolated from GPX3^fl/fl^Alb^cre^ and GPX3^fl/fl^ mice, n=3. For experiments involving genotype/treatment and time, statistical significance was determined by two-way ANOVA followed by Tukey’s multiple comparisons test. ***p <0.001

**Figure S4**


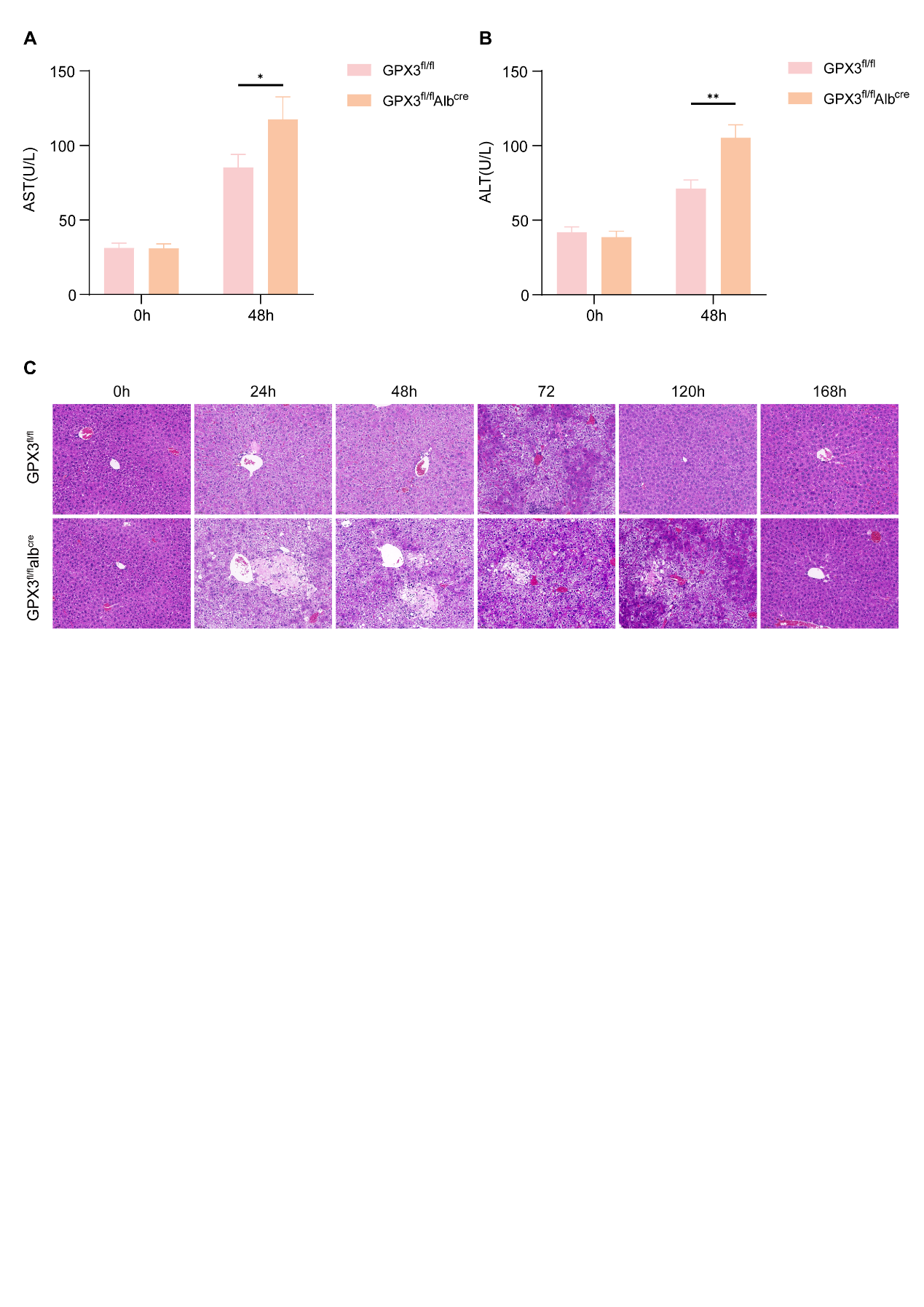


**Figure S4. Hepatocyte-specific GPX3 deficiency impairs liver regeneration following PH.** (A, B) ALT and AST levels in GPX3^fl/fl^Alb^cre^ and GPX3^fl/fl^ mice at indicated time points post-PH, n=3. (C) Representative H&E staining in liver tissues at indicated time points post-PH (scale bar=100μm), n=3. For experiments involving genotype/treatment and time, statistical significance was determined by two-way ANOVA followed by Tukey’s multiple comparisons test. *p <0.05, **p <0.01

**Figure S5**

**
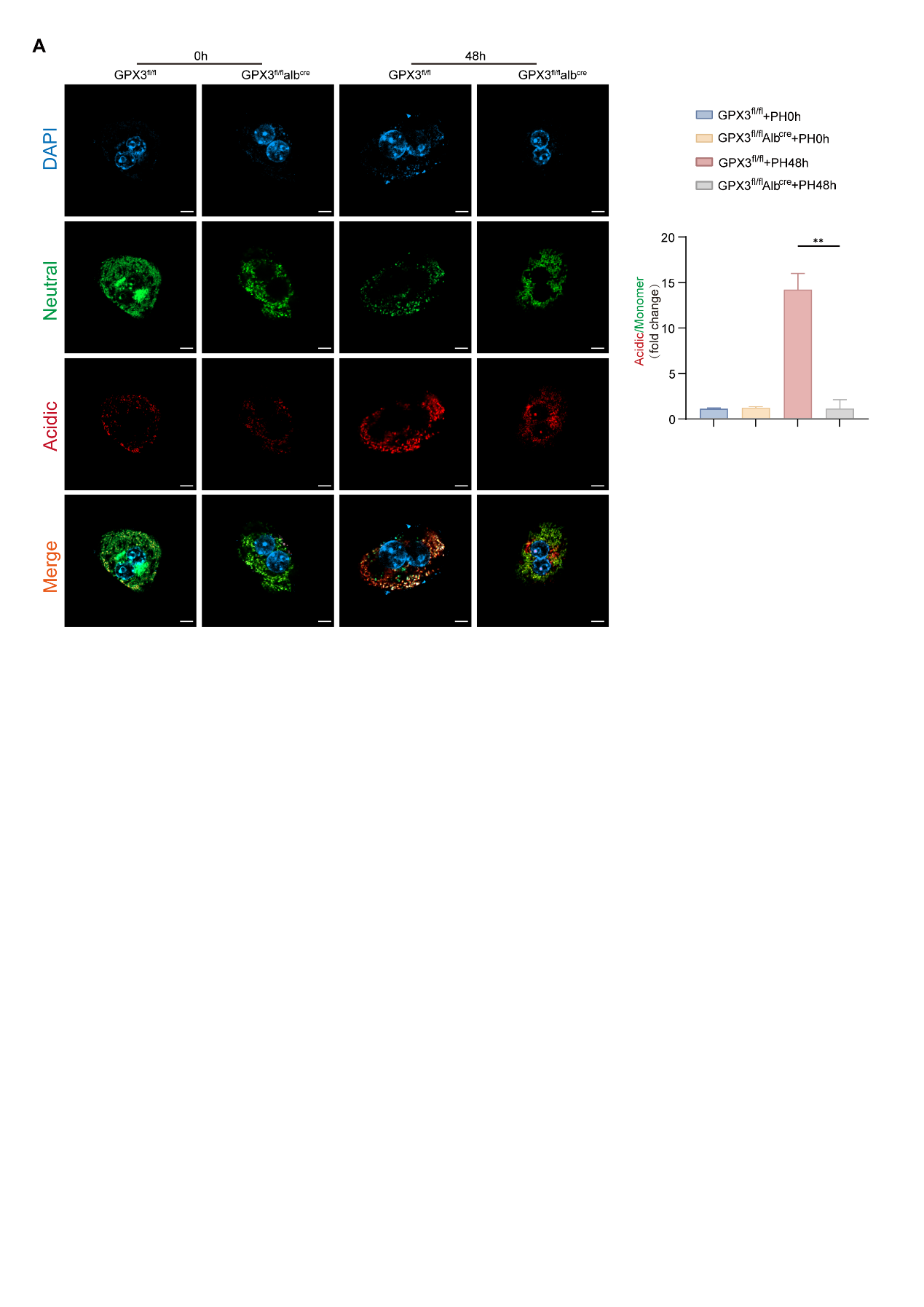
**

**Figure S5. Hepatocyte-specific GPX3 deficiency impairs mitophagy during liver regeneration.** (A) Representative images of Lv-mtKeima-COX8 fluorescence in primary hepatocytes isolated from GPX3^fl/fl^Alb^cre^ and GPX3^fl/fl^ mice at 0 h and 48 h after partial hepatectomy, n=3 (scale bar=10μm). For experiments involving genotype/treatment and time, statistical significance was determined by two-way ANOVA followed by Tukey’s multiple comparisons test. **p <0.01

**Figure S6**


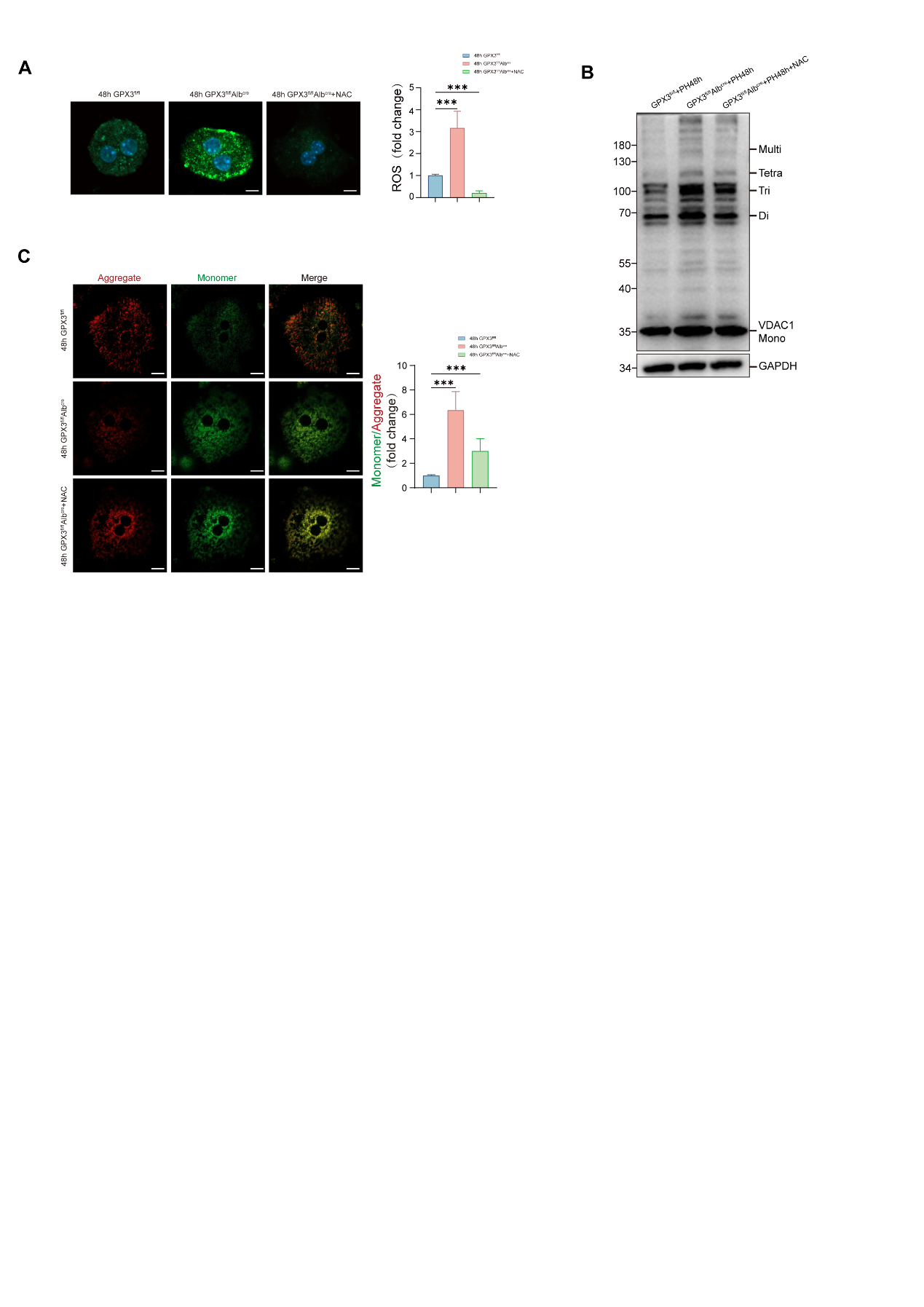


**Figure S6.  GPX3 deficiency-induced VDAC1 oligomerization is not fully dependent on its antioxidant activity.** (A) DCFH-DA fluorescence imaging of primary hepatocytes showing ROS levels in primary hepatocytes following NAC treatment, n=3 (scale bar=10μm). (B) Western blot analysis of VDAC1 oligomerization in NAC-treated and control mice, n=3. (C) Representative JC-10-stained images and quantification of mitochondria depolarization in primary hepatocytes following NAC treatment, n=3 (scale bar=10μm). For experiments involving genotype/treatment and time, statistical significance was determined by two-way ANOVA followed by Tukey’s multiple comparisons test. ***p <0.001

**Figure S7**

**
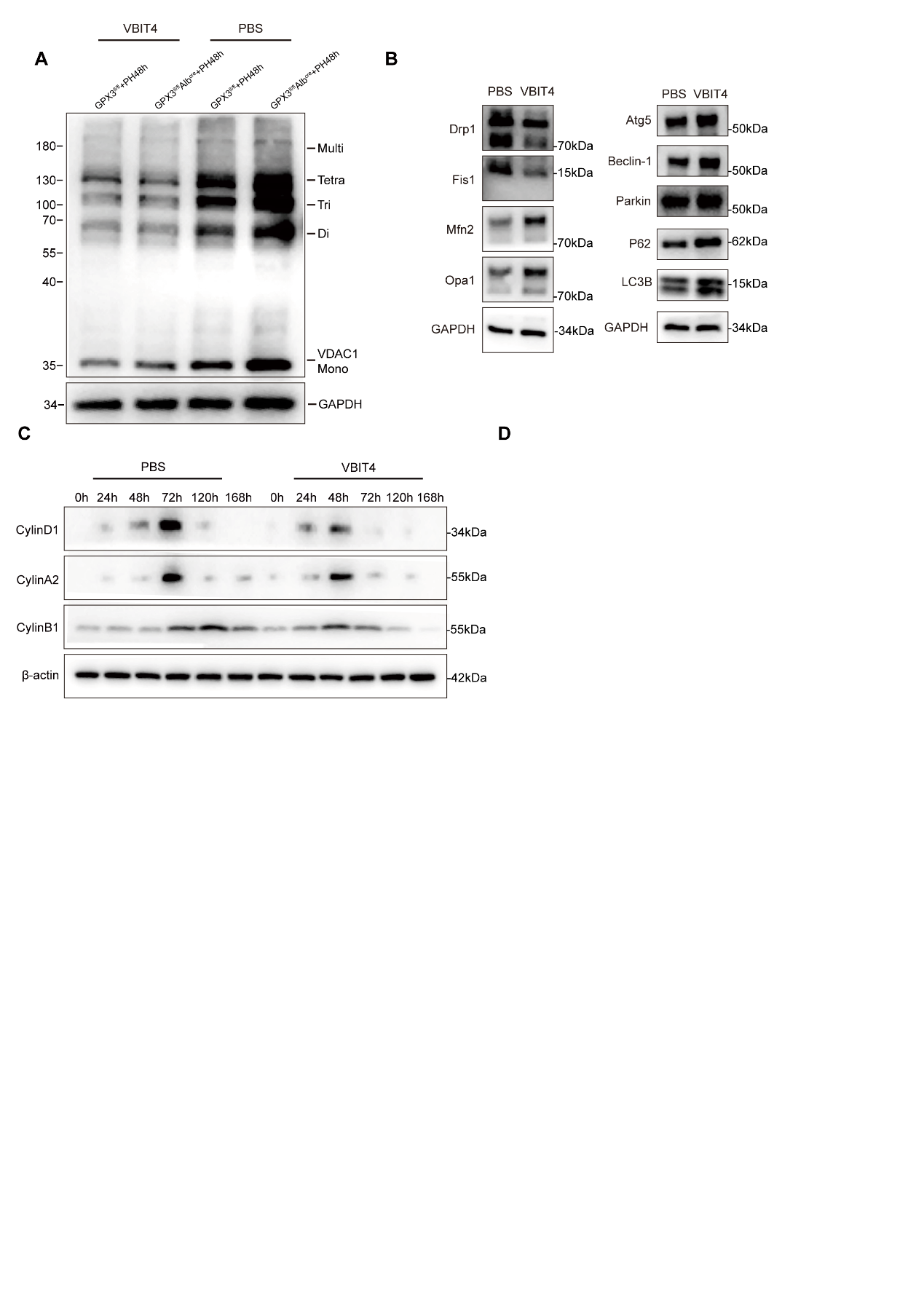
**

**Figure S7. VDAC1 Oligomerization is Associated with Mitochondrial Function and Liver Regeneration.** (A) Western blot analysis of VDAC1 oligomerization in VBIT-4-treated and control mice, n=3. (B) Western blot analysis of MQC related proteins in VBIT-4-treated and control mice, n=3. (C) Western blot analysis of cell cycle markers in VBIT-4-treated and control mice, n=3. For experiments involving genotype/treatment and time, statistical significance was determined by two-way ANOVA followed by Tukey’s multiple comparisons test. *p <0.05, **p <0.01, *p <0.001

**
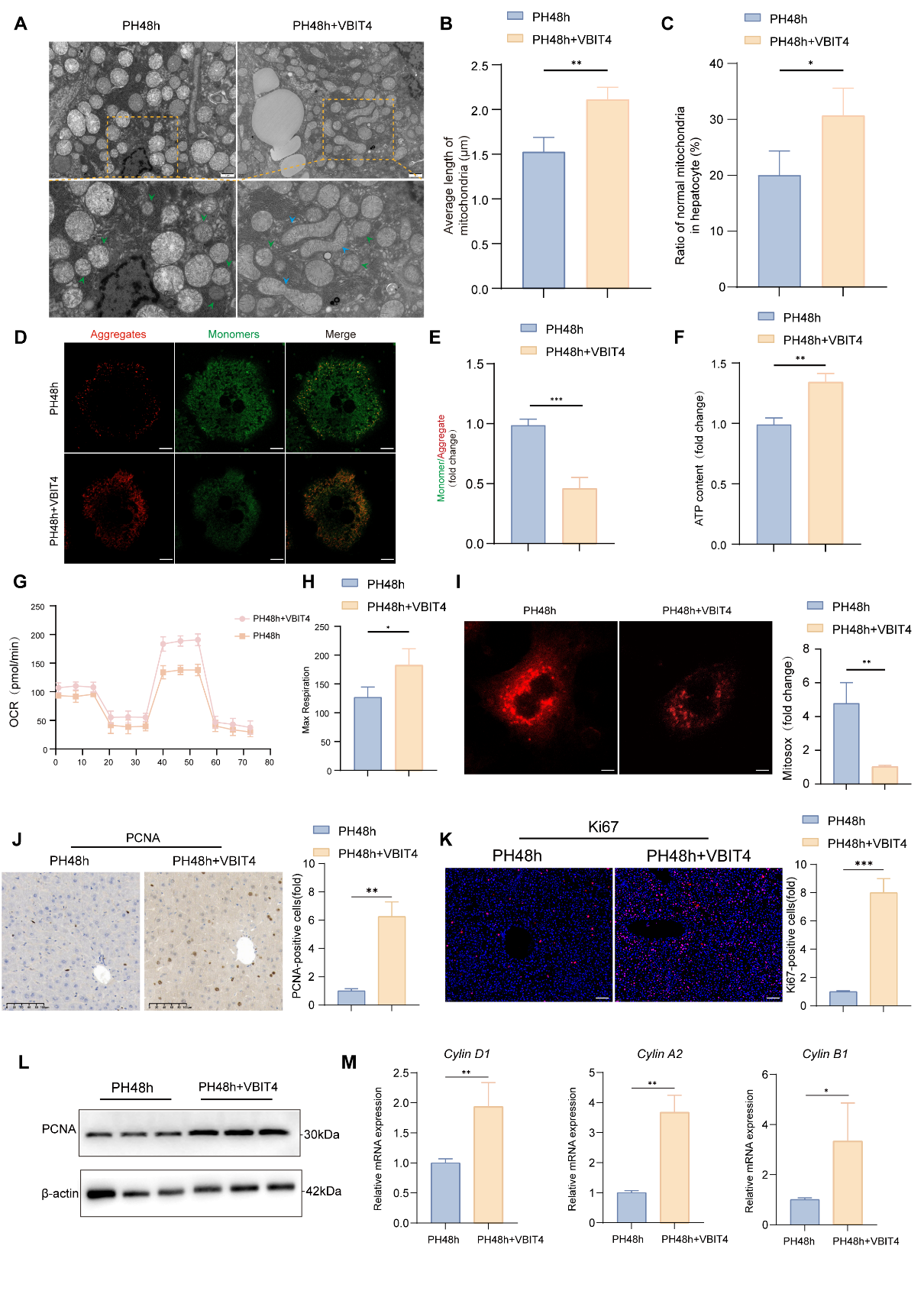
Figure S8**

**Figure S8 Suppression of VDAC1 oligomerization abrogated the detrimental effects of hepatocyte GPX3 depletion during liver regeneration.**

(A) TEM (scale bar=2μm) visualization of hepatocyte mitochondria at 48h post-PH following VIBT4 treatment. (B, C) Average mitochondrial length and ratio of fragmented/tubular mitochondria were determined, n=5. (D, E) Representative JC-10-stained images (D) and quantification of mitochondria depolarization (E) in primary hepatocytes following VIBT4 treatment, n=3 (scale bar=10μm). (F) ATP content in primary hepatocytes isolated from GPX3^fl/fl^Alb^cre^ at 48h post-PH following VIBT4 treatment, n=3.

(G, H) OCR measurement in primary hepatocytes isolated from GPX3^fl/fl^Alb^cre^ mice at 48h post-PH following VIBT4 treatment, and the analysis of mitochondrial respiration, n=5. (I) MitoSOX Red fluorescence indicating mitochondrial superoxide production at 48h post-PH following VIBT4 treatment (scale bar=10μm). Quantified fold change of MitoSOX signal intensity on the right, n=3. (J) PCNA immunohistochemical staining in GPX3^fl/fl^Alb^cre^ at 48h post-PH following VIBT4 treatment. Quantification of PCNA-positive cells is shown on the right (scale bar=100μm). (K) Representative immunofluorescence staining of Ki67 in GPX3^fl/fl^Alb^cre^ at 48h post-PH following VIBT4 treatment. Quantification of Ki67-positive cells is shown on the right (scale bar=100μm). (L) Western blot analysis of PCNA in GPX3^fl/fl^Alb^cre^ at 48h post-PH following VIBT4 treatment, n=3. (M) qPCR analysis of proliferation-related genes (CyclinD1, CyclinA2 and CyclinB1) expression in GPX3^fl/fl^Alb^cre^ at 48h post-PH following VIBT4 treatment, n=3. For experiments involving genotype/treatment and time, statistical significance was determined by two-way ANOVA followed by Tukey’s multiple comparisons test. *p <0.05, **p <0.01, ***p <0.001

**Figure S9**

**
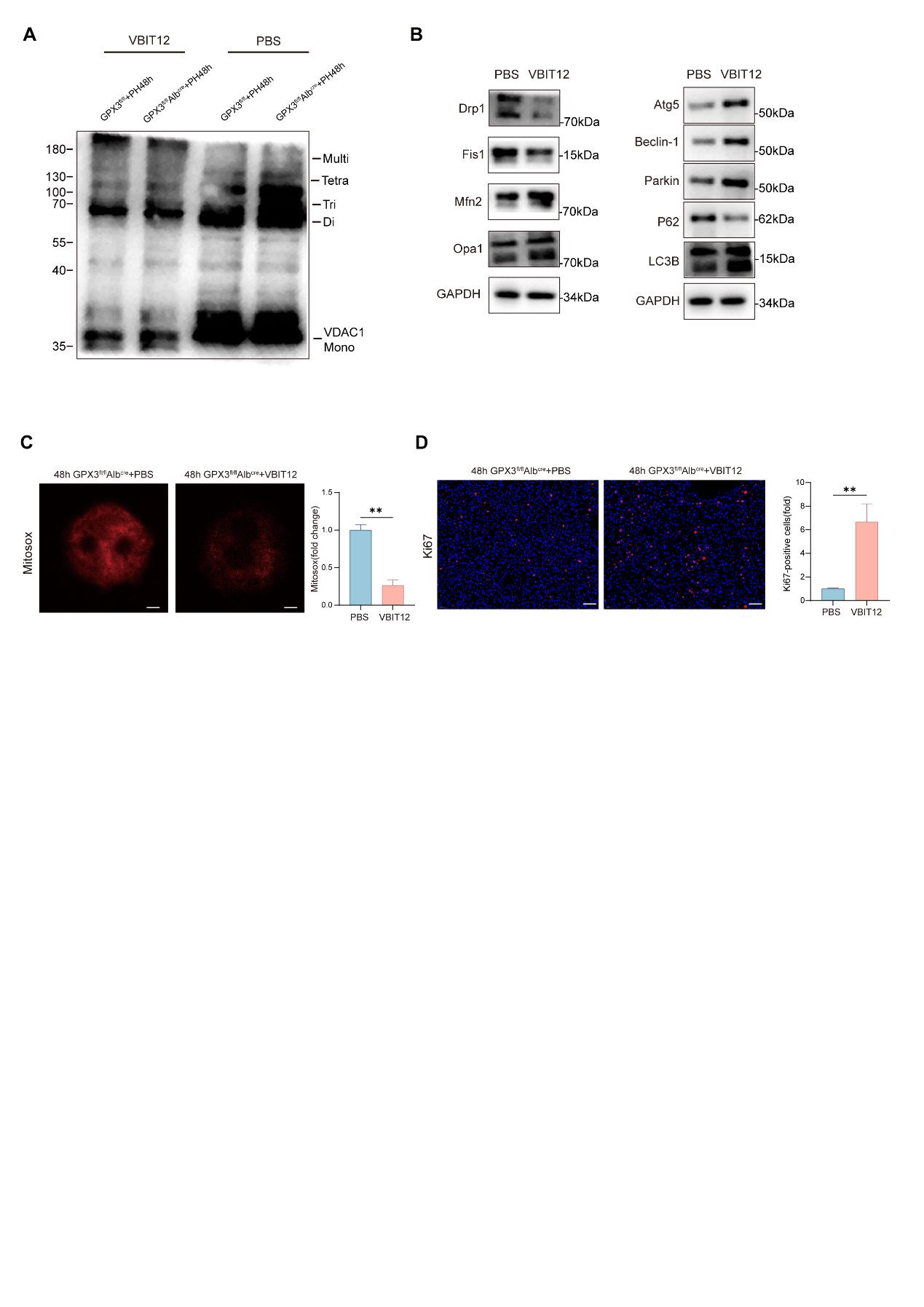
**

**Figure S9 VBIT-12 alleviates GPX3 deficiency-induced adverse phenotypes by inhibiting VDAC1 oligomerization**

(A) Western blot analysis of VDAC1 oligomerization in VBIT-12-treated and control mice, n=3. (B) Western blot analysis of MQC related proteins in VBIT-12-treated and control mice, n=3. (C) MitoSOX Red fluorescence indicating mitochondrial superoxide production at 48h post-PH following VIBT12 treatment (scale bar=10μm). Quantified fold change of MitoSOX signal intensity on the right, n=3. (D) Representative immunofluorescence staining of Ki67 in GPX3^fl/fl^Alb^cre^ at 48h post-PH following VIBT12 treatment. Quantification of Ki67-positive cells is shown on the right (scale bar=100μm). For experiments involving genotype/treatment and time, statistical significance was determined by two-way ANOVA followed by Tukey’s multiple comparisons test. **p <0.01

**Figure S10**


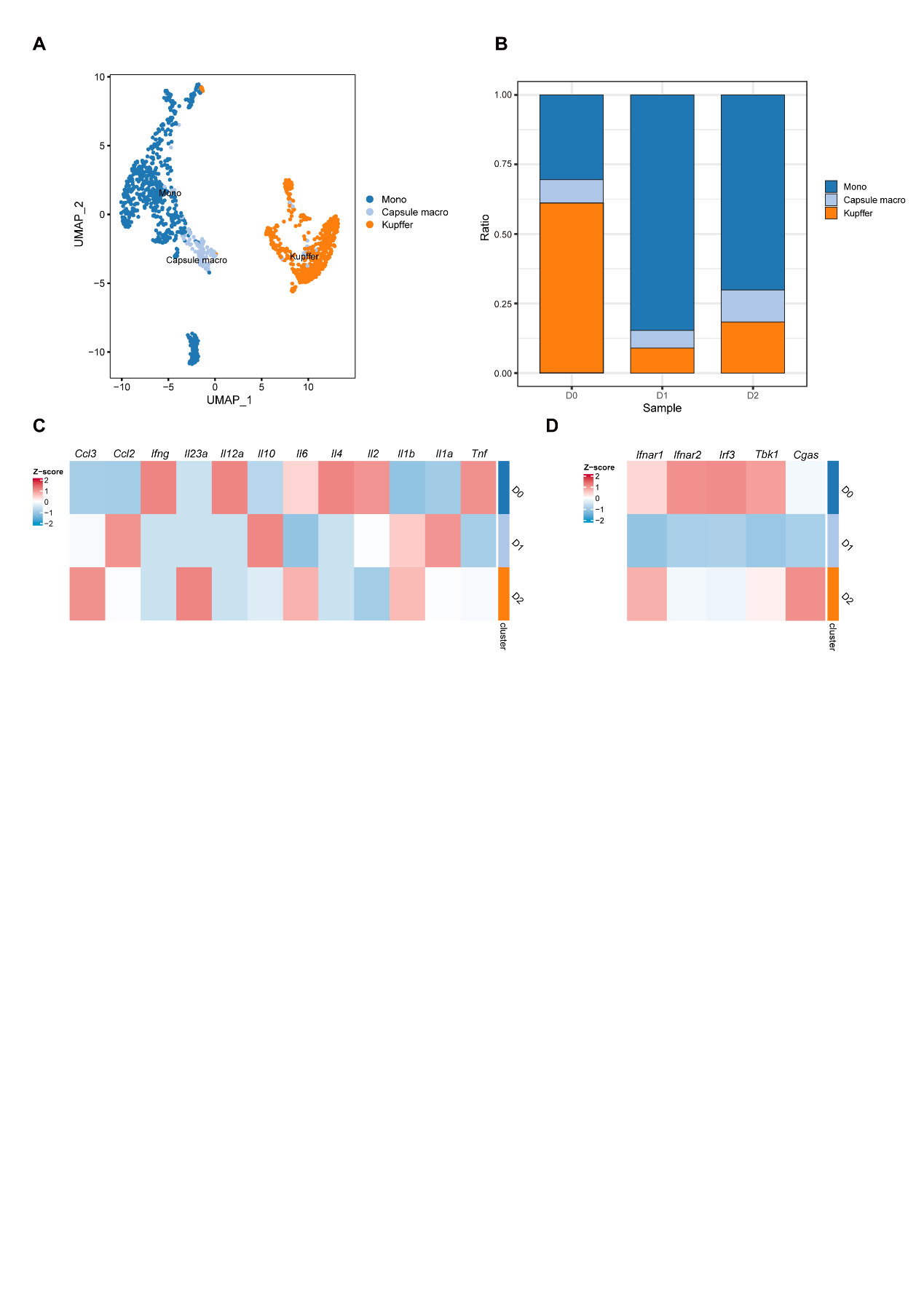


**Figure S10 Single-cell analysis reveals dynamic remodeling of macrophage subsets during liver regeneration.**

(A) UMAP plot showing hepatic macrophage populations identified in the public scRNA-seq dataset, including monocytes (Mono), capsule macrophages (Capsule macro) and Kupffer cells. (B) Relative proportions of these populations at D0, D1 and D2 after partial hepatectomy. (C, D) Heatmaps showing the expression of representative inflammatory genes (C) and STING/interferon-related genes (D) across the indicated macrophage/monocyte subsets. For experiments involving genotype/treatment and time, statistical significance was determined by two-way ANOVA followed by Tukey’s multiple comparisons test.


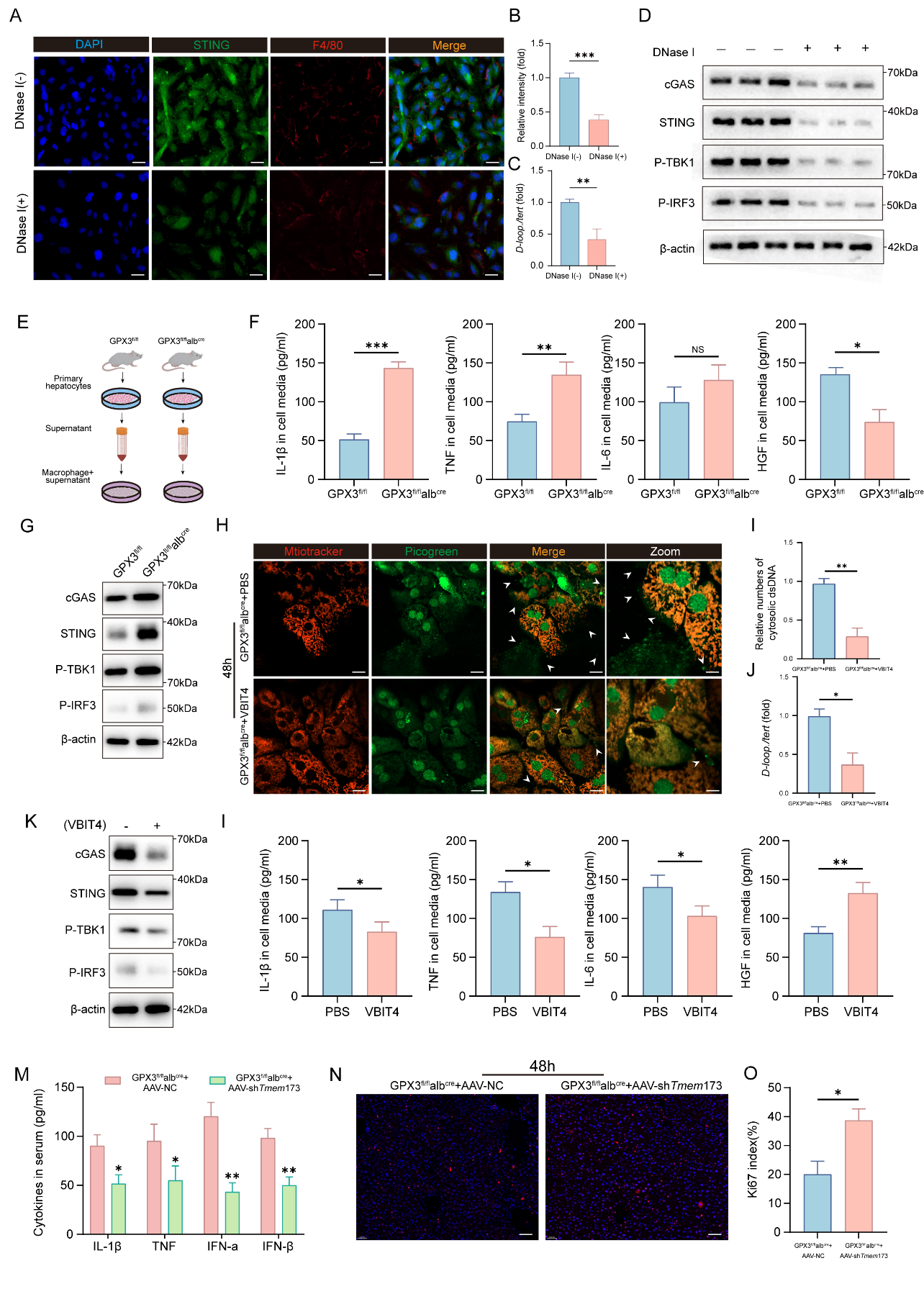
**Figure S11.**

**Figure S11. VDAC1 oligomerization induces hepatocyte mtDNA release and activates cGAS–STING pathway in macrophages**

(A) Intrahepatic macrophages were isolated from livers post PH of control and DNase I-treated mice, followed by staining of STING (red), F4/80 (green), and nucleus (blue). (scale bar = 100 μm). (B) Quantified fold change of STING signal intensity, n=3. (C) Relative amounts of total cytosolic mtDNA in macrophages isolated from control and DNase I-treated mice were determined using qPCR with primers specific for mtDNA (D-loop) and nuclear DNA (Tert), n = 3. (D) Western blot analysis of cGAS, STING, p-TBK1 and p-IRF3, n=3. (E) Schematic illustration of macrophage stimulation by primary hepatocyte supernatant. (F) ELISA analysis of IL-1β, TNF-a, IL-6, and HGF secreted by macrophages from the co-culture system. (G) Western blot analysis of cGAS, STING, p-TBK1, and p-IRF3 in macrophages from the co-culture system, n=3. (H, I) MtDNA released from mitochondria in GPX3^fl/fl^Alb^cre^+PBS and GPX3^fl/fl^Alb^cre^+VBIT-4 post PH, as shown by confocal microscopy. Arrowheads, mtDNA released into cytoplasm (scale bar=100μm). (J) Relative amounts of total cytosolic mtDNA in macrophages, n = 3. (K) Western blot analysis of cGAS, STING, p-TBK1 and p-IRF3 in macrophages from VBIT-4-treated and control mice, n=3. (I) Serum IL-1β, TNF-a, IFN-a, and HGF levels assayed by ELISA from VBIT-4-treated and control mice, n=3. (M) Serum IL-1β, TNF-a, IFN-a, and IFN-β levels assayed by ELISA in GPX3^fl/fl^Alb^cre^ +AAV-Vector and GPX3^fl/fl^Alb^cre^ +AAV-shTmem173 mice post PH, n=3. (N, O) Representative Ki67 immunohistochemical staining and quantification in GPX3^fl/fl^Alb^cre^ +AAV-Vector and GPX3^fl/fl^Alb^cre^ +AAV-shTmem173 mice. For experiments involving genotype/treatment and time, statistical significance was determined by two-way ANOVA followed by Tukey’s multiple comparisons test. NS p>0.05, *p <0.05, **p <0.01, ***p <0.001

**Figure S12**


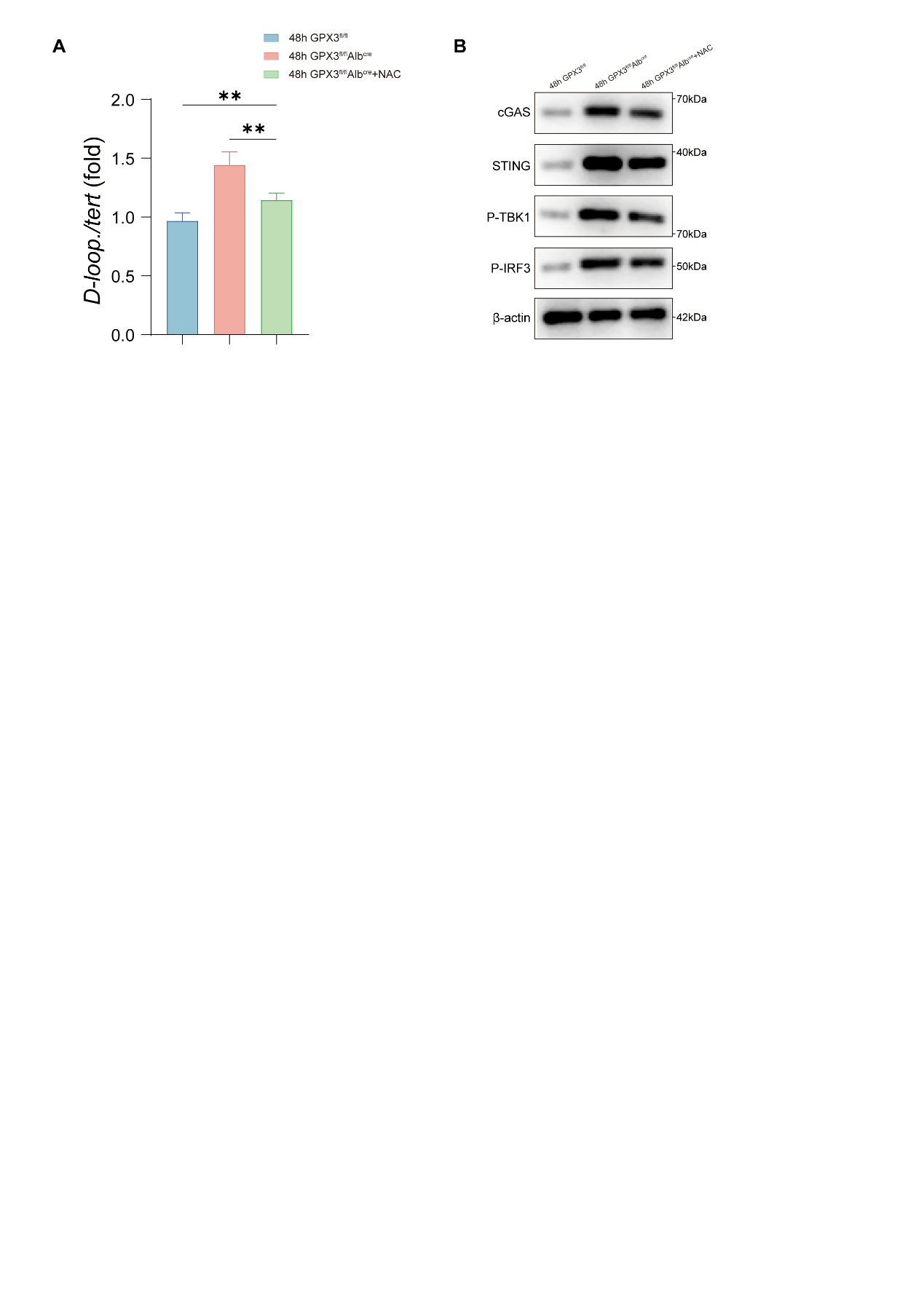


**Figure S12 GPX3 deficiency-induced mtDNA release and STING activation are not fully dependent on its antioxidant activity.**

(A) Relative amounts of total cytosolic mtDNA in macrophages isolated from control and NAC-treated mice were determined using qPCR with primers specific for mtDNA (D-loop) and nuclear DNA (Tert), n = 3. (B) Western blot analysis of cGAS, STING, p-TBK1 and p-IRF3 in macrophages from NAC-treated and control mice, n=3. For experiments involving genotype/treatment and time, statistical significance was determined by two-way ANOVA followed by Tukey’s multiple comparisons test. **p <0.01

**Figure S13**

**
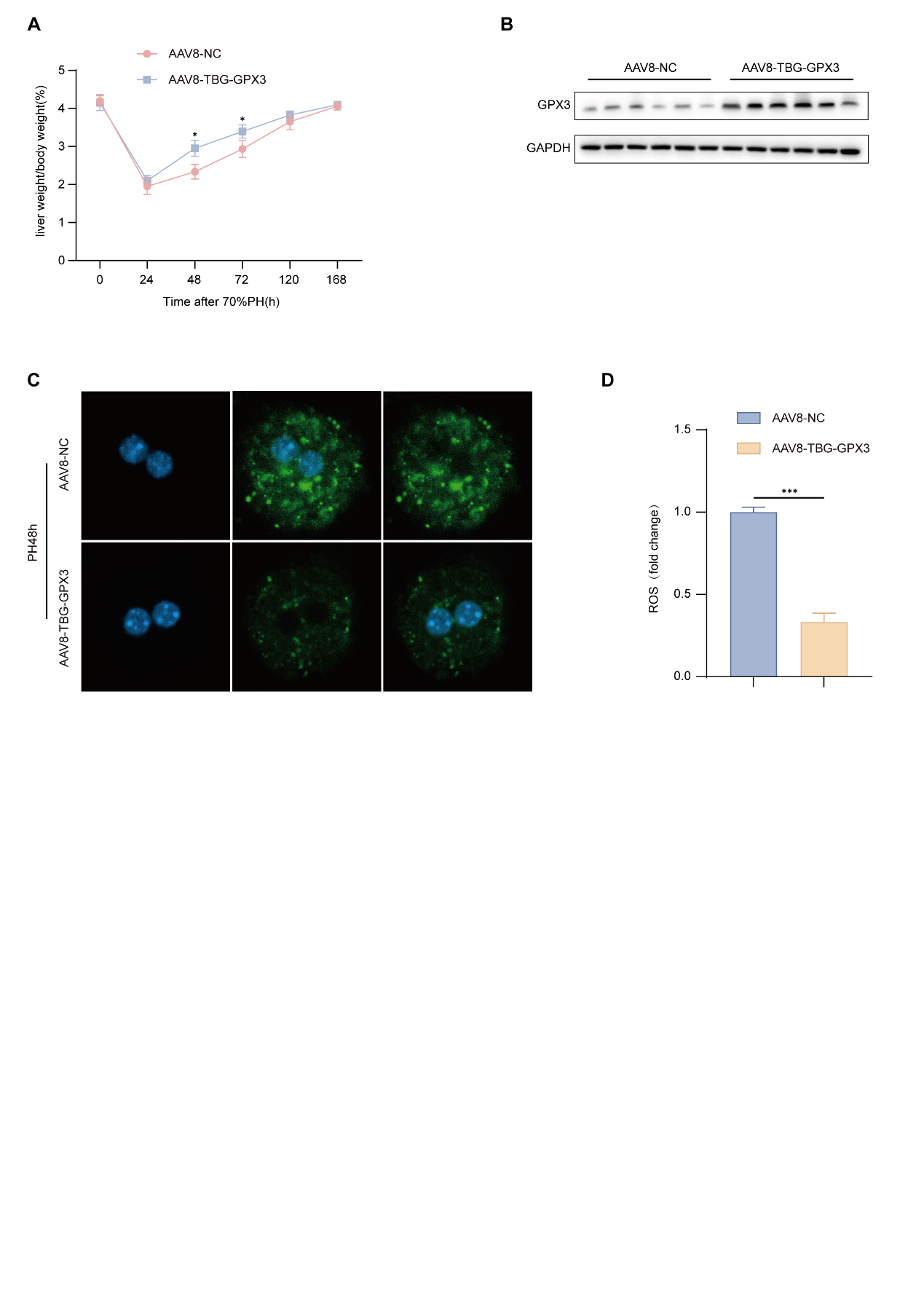
Figure S13 Hepatocyte-specific overexpression of GPX3 promotes liver regeneration.** (A) The ratios of liver weight/body weight at different time points after PH. (B) Western blot analysis and quantitation of GPX3 in primary hepatocytes after rejectingAAV8, n=6. (C) DCFH-DA fluorescence imaging of primary hepatocytes showing ROS levels at 48h post-PH in AAV8-TBG-GPX3 and AAV8-NC mice (scale bar=10μm), n=3. (D) Quantified ROS levels expressed as fold change relative to 0h control, n=3. For experiments involving genotype/treatment and time, statistical significance was determined by two-way ANOVA followed by Tukey’s multiple comparisons test. ***p <0.001

**Figure S14**


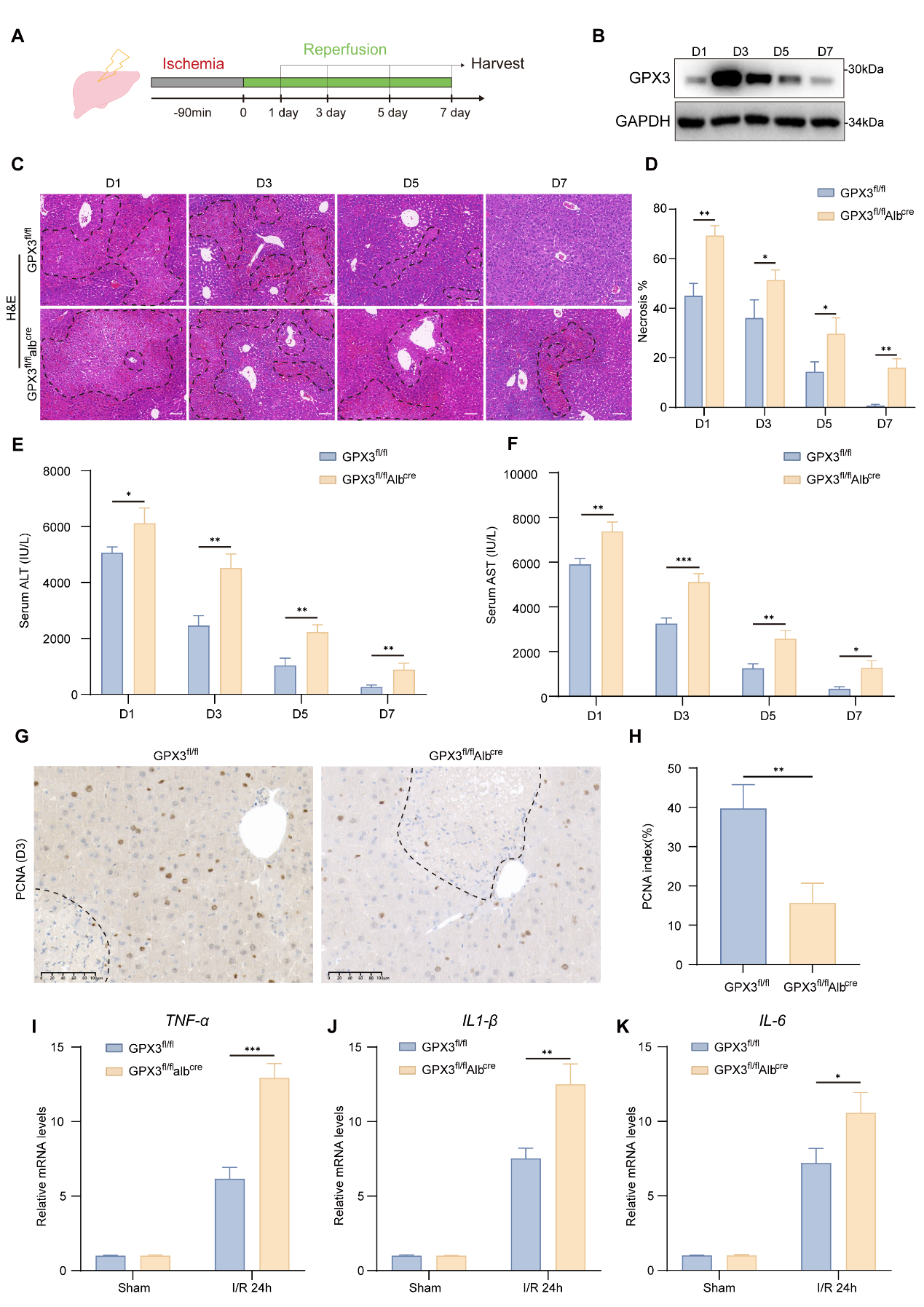


**Figure S14 GPX3 deficiency in hepatocytes delays liver regeneration following ischemia-reperfusion injury.**

(A) Schematic overview of HIRI model. (B) Western blot analysis and quantitation of GPX3 in primary hepatocytes at indicated time points after reperfusion following 90-min ischemia. (C, D) Representative H&E staining and necrotic area quantification in liver tissues after HIRI. Dashed lines demarcate necrotic areas (scale bar=100μm), n=3. (E, F) ALT and AST levels in GPX3^fl/fl^Alb^cre^ and GPX3^fl/fl^ mice after HIRI, n=3. (G, H) PCNA immunohistochemical staining in GPX3^fl/fl^Alb^cre^ and GPX3^fl/fl^ mice at 3day after reperfusion, with corresponding quantitative analysis shown on the right (scale bar=100μm), n=3. (I-K) RNA levels of TNF-α, IL1-β, IL-6 measured by qRT-PCR, n=5. For experiments involving genotype/treatment and time, statistical significance was determined by two-way ANOVA followed by Tukey’s multiple comparisons test. *p <0.05, **p <0.01, ***p <0.001

**Supplementary Table 1: Antibodies used in this study**

| Reagent or Resource | Source | Cat no. |
| --- | --- | --- |
| Antibodies |  |  |
| anti-GPX3 | Abcam | Cat#ab256470 |
|  | Proteintech | Cat#13947-1-AP |
| anti-CylinD1 | Cell Signaling Technology | Cat# 55506T |
| anti-CylinA2 | Abcam | Cat# ab181591 |
| anti-CylinB1 | Cell Signaling Technology | Cat# 4138T |
| anti-Drp1 | Proteintech | Cat#12957-1-AP |
| anti-Fis1 | Proteintech | Cat# 10956-1-AP |
| anti-Opa1 | Cell Signaling Technology | Cat# 80471T |
| anti-Atg5 | Abcam | Cat# ab108327 |
| anti-Beclin-1 | Proteintech | Cat#11306-1-AP |
| anti-Parkin | Abcam | Cat#ab77924 |
|  | Proteintech | Cat#14060-1-AP |
| anti-Flag | MBL | Cat#M185-3 |
| anti-His | MBL | Cat#D291-3 |
| anti-VDAC1 | Proteintech | Cat# 55259-1-AP |
|  | Cell Signaling Technology | Cat# 4866T |
|  | Cell Signaling Technology | Cat#3933 |
| anti-cGAS | Cell Signaling Technology | Cat #31659S |
| anti-STING | Cell Signaling Technology | Cat #13647 |
| anti-P-TBK1 | Cell Signaling Technology | Cat #5483S |
| anti-P-IRF3 | Cell Signaling Technology | Cat #29047S |
| Goat anti-mouse IgG (H+L) | Jackson | Cat#115-035-003 |
| Goat anti-rabbit IgG (H+L) | Jackson | Cat#111-035-003 |
| Alexa Flour 568 goat anti-rabbit IgG (H+L) | Invitrogen | Cat#A11036 |
| Alexa Flour 488 goat anti-mouse IgG (H+L) | Invitrogen | Cat#A11029 |
| anti-β-actin | Abclonal | Cat#AC026 |
| anti-GAPDH | Proteintech | Cat#60004-1-Ig |

**Supplementary Table 2: Primers sequences used for real-time PCR**

| Gene | Species | Primer Sequence(5'-3') |
| --- | --- | --- |
| GPX3 | Mouse | Forward: AGCTAGTCCAGCGTAATAGGGAGG |
|  |  | Reverse: GGATGTTGGGAATGAGTCAGAGC |
| VDAC1 | Mouse | Forward: ACGTATGCCGATCTTGGCAAA |
|  |  | Reverse: TCAGGCCGTACTCAGTCCATC |
| CylinD1 | Mouse | Forward: GCGTACCCTGACACCAATCTC |
|  |  | Reverse: CTCCTCTTCGCACTTCTGCTC |
| CylinA2 | Mouse | Forward: ACAGAGTGTGAAGATGCCCTGGCT |
|  |  | Reverse: AGCATGTGGTGATTCAAAACTGCCA |
| CylinB1 | Mouse | Forward: AAGGTGCCTGTGTGTGAACC |
|  |  | Reverse: GTCAGCCCCATCATCTGCG |

**REFERENCES**

1. Mitchell C, Willenbring H. A reproducible and well-tolerated method for 2/3 partial hepatectomy in mice. Nature protocols. 2008;3(7):1167-70.

2. Zhou H, Wang H, Ni M, Yue S, Xia Y, Busuttil RW, et al. Glycogen synthase kinase 3β promotes liver innate immune activation by restraining AMP-activated protein kinase activation. Journal of hepatology. 2018;69(1):99-109.

3. Xu J, Guo P, Hao S, Shangguan S, Shi Q, Volpe G, et al. A spatiotemporal atlas of mouse liver homeostasis and regeneration. Nature genetics. 2024;56(5):953-69.

4. Yang J, Lu X, Hao JL, Li L, Ruan YT, An XN, et al. VSTM2L protects prostate cancer cells against ferroptosis via inhibiting VDAC1 oligomerization and maintaining mitochondria homeostasis. Nature communications. 2025;16(1):1160.

5. Kim J, Gupta R, Blanco LP, Yang S, Shteinfer-Kuzmine A, Wang K, et al. VDAC oligomers form mitochondrial pores to release mtDNA fragments and promote lupus-like disease. Science (New York, NY). 2019;366(6472):1531-6.

6. Yao F, Zhou S, Zhang R, Chen Y, Huang W, Yu K, et al. CRISPR/Cas9 screen reveals that targeting TRIM34 enhances ferroptosis sensitivity and augments immunotherapy efficacy in hepatocellular carcinoma. Cancer letters. 2024;593:216935.

7. Wiśniewski JR, Zougman A, Nagaraj N, Mann M. Universal sample preparation method for proteome analysis. Nature methods. 2009;6(5):359-62.
