# Supplementary material for: Glutathione peroxidase 3 preserves hepatocyte mitochondrial quality control to enhance macrophage pro‐regenerative phenotype during liver regeneration
Source: Clin Transl Med. 2026 May 29;16(6):e70695. doi: 10.1002/ctm2.70695 (PMC13240042; doi:10.1002/ctm2.70695)
Supplement: Supplementary file 2 — SUPPORTING INFORMATION [file CTM2-16-e70695-s002.docx]

**Supplementary Table 1: Antibodies used in this study**

| Reagent or Resource | Source | Cat no. |
| --- | --- | --- |
| Antibodies |  |  |
| anti-GPX3 | Abcam | Cat#ab256470 |
|  | Proteintech | Cat#13947-1-AP |
| anti-CylinD1 | Cell Signaling Technology | Cat# 55506T |
| anti-CylinA2 | Abcam | Cat# ab181591 |
| anti-CylinB1 | Cell Signaling Technology | Cat# 4138T |
| anti-Drp1 | Proteintech | Cat#12957-1-AP |
| anti-Fis1 | Proteintech | Cat# 10956-1-AP |
| anti-Opa1 | Cell Signaling Technology | Cat# 80471T |
| anti-Atg5 | Abcam | Cat# ab108327 |
| anti-Beclin-1 | Proteintech | Cat#11306-1-AP |
| anti-Parkin | Abcam | Cat#ab77924 |
|  | Proteintech | Cat#14060-1-AP |
| anti-Flag | MBL | Cat#M185-3 |
| anti-His | MBL | Cat#D291-3 |
| anti-VDAC1 | Proteintech | Cat# 55259-1-AP |
|  | Cell Signaling Technology | Cat# 4866T |
|  | Cell Signaling Technology | Cat#3933 |
| anti-cGAS | Cell Signaling Technology | Cat #31659S |
| anti-STING | Cell Signaling Technology | Cat #13647 |
| anti-P-TBK1 | Cell Signaling Technology | Cat #5483S |
| anti-P-IRF3 | Cell Signaling Technology | Cat #29047S |
| Goat anti-mouse IgG (H+L) | Jackson | Cat#115-035-003 |
| Goat anti-rabbit IgG (H+L) | Jackson | Cat#111-035-003 |
| Alexa Flour 568 goat anti-rabbit IgG (H+L) | Invitrogen | Cat#A11036 |
| Alexa Flour 488 goat anti-mouse IgG (H+L) | Invitrogen | Cat#A11029 |
| anti-β-actin | Abclonal | Cat#AC026 |
| anti-GAPDH | Proteintech | Cat#60004-1-Ig |

**Supplementary Table 2: Primers sequences used for real-time PCR**

| Gene | Species | Primer Sequence(5'-3') |
| --- | --- | --- |
| GPX3 | Mouse | Forward: AGCTAGTCCAGCGTAATAGGGAGG |
|  |  | Reverse: GGATGTTGGGAATGAGTCAGAGC |
| VDAC1 | Mouse | Forward: ACGTATGCCGATCTTGGCAAA |
|  |  | Reverse: TCAGGCCGTACTCAGTCCATC |
| CylinD1 | Mouse | Forward: GCGTACCCTGACACCAATCTC |
|  |  | Reverse: CTCCTCTTCGCACTTCTGCTC |
| CylinA2 | Mouse | Forward: ACAGAGTGTGAAGATGCCCTGGCT |
|  |  | Reverse: AGCATGTGGTGATTCAAAACTGCCA |
| CylinB1 | Mouse | Forward: AAGGTGCCTGTGTGTGAACC |
|  |  | Reverse: GTCAGCCCCATCATCTGCG |
